# Medicare Eligibility and Changes in Coverage, Access to Care, and Health by Sexual Orientation and Gender Identity

**DOI:** 10.1001/jamahealthforum.2024.1756

**Published:** 2024-07-05

**Authors:** Kyle A. Gavulic, Jacob Wallace

**Affiliations:** 1 Yale School of Medicine, New Haven, Connecticut; 2 Department of Health Policy and Management, Yale School of Public Health, New Haven, Connecticut

## Abstract

**Importance:**

Medicare provides nearly universal insurance coverage at age 65 years. However, how Medicare eligibility affects disparities in health insurance coverage, access to care, and health status among individuals by sexual orientation and gender identity is poorly understood.

**Objective:**

To assess the association of Medicare eligibility with disparities in health insurance coverage, access to care, and self-reported health status among individuals by sexual orientation and by gender identity.

**Design, Setting, and Participants:**

This cross-sectional study used the age discontinuity for Medicare eligibility at age 65 years to isolate the association of Medicare with health insurance coverage, access to care, and self-reported health status, by their sexual orientation and by their gender identity. Data were collected from the Behavioral Risk Factor Surveillance System for respondents from 51 to 79 years old from 2014 to 2021. Data analysis was performed from September 2022 to April 2023.

**Exposures:**

Medicare eligibility at age 65 years.

**Main Outcomes and Measures:**

Proportions of respondents with health insurance coverage, usual source of care, cost barriers to care, influenza vaccination, and self-reported health status.

**Results:**

The study population included 927 952 individuals (mean [SD] age, 64.4 [7.7] years; 524 972 [56.6%] females and 402 670 [43.4%] males), of whom 28 077 (3.03%) identified as a sexual minority—lesbian, gay, bisexual, or another sexual minority identity (LGB+) and 3286 (0.35%) as transgender or gender diverse. Respondents who identified as heterosexual had greater improvements at age 65 years in insurance coverage (4.2 percentage points [pp]; 95% CI, 4.0-4.4 pp) than those who identified as LGB+ (3.6 pp; 95% CI, 2.3-4.8 pp), except when the analysis was limited to a subsample of married respondents. For access to care, improvements in usual source of care, cost barriers to care, and influenza vaccination were larger at age 65 years for heterosexual respondents compared with LGB+ respondents, although confidence intervals were overlapping and less precise for LGB+ individuals. For self-reported health status, the analyses found larger improvements at age 65 years for LGB+ respondents compared with heterosexual respondents. There was considerable heterogeneity by state in disparities by sexual orientation among individuals who were nearly eligible for Medicare (close to 65 years old), with the US South and Central states demonstrating the highest disparities. Among the top-10 highest-disparities states, Medicare eligibility was associated with greater increases in coverage (6.7 pp vs 5.0 pp) and access to a usual source of care (1.4 pp vs 0.6 pp) for LGB+ respondents compared with heterosexual respondents.

**Conclusions and Relevance:**

The findings of this cross-sectional study indicate that Medicare eligibility was not associated with consistently greater improvements in health insurance coverage and access to care among LGBTQI+ individuals compared with heterosexual and/or cisgender individuals. However, among sexual minority individuals, Medicare may be associated with closing gaps in self-reported health status, and among states with the highest disparities, it may improve health insurance coverage, access to care, and self-reported health status.

## Key Points

**Question:**

Is Medicare eligibility associated with reduced disparities in health insurance coverage, access to care, and self-reported health status among individuals by sexual orientation or gender identity?

**Findings:**

This cross-sectional study using regression discontinuity found that Medicare eligibility at age 65 years was associated with larger increases in self-reported health status among respondents identifying as lesbian, gay, bisexual or another sexual minority identity (LGB+) than those identifying as heterosexual, but smaller increases in insurance coverage and access to care. Yet in the US states with the highest preexisting disparities, LGB+ individuals reported larger improvements in insurance coverage, access, and health status than did heterosexual individuals.

**Meaning:**

These findings indicate that Medicare eligibility is associated with improved self-reported health among LGB+ adults compared with heterosexual adults; additionally, in states with larger disparities, Medicare may reduce disparities in insurance coverage and access to care between the groups.

## Teaser

This cross-sectional study assesses the association of Medicare with disparities in health insurance coverage, access to care, and self-reported health status among individuals by sexual orientation and by gender identity.

## Introduction

Stigma, structural violence, and discrimination propagated by institutions, systems, and policies have often pushed persons who identify as lesbian, gay, bisexual, transgender, queer, intersex, or another sexual or gender minority (LGBTQI+) to the margins of society. Accordingly, those who identify as LGBTQI+ experience worse access to health care,^1,2,3,4,5^ greater financial burden of health care spending,^6^ worse health outcomes, including a greater likelihood of developing a chronic condition^3,4,5,7,8,9,10^ and historically higher rates of uninsurance.^1,3,4,11,12,13,14,15^

Recognizing the inequities faced by LGBTQI+ populations, the Biden Administration published the *Federal Evidence Agenda on LGBTQI+ Equity* in January 2023 to call for improved sexual orientation and gender identity (SOGI) data collection and evidence generation to “advance equity for and improve the well-being of LGBTQI+ people.”^16^ To promote more equitable access to health care in the US, some federal policymakers have proposed lowering the Medicare eligibility age.^17^ However, while historical LGBTQI+ disparities in coverage are well documented, it is unclear whether expanding Medicare eligibility would meaningfully reduce disparities in health insurance coverage, access to care, and health status among individuals by sexual orientation and by gender identity.

Prior research shows that Medicare eligibility improves access to care,^18,19,20,21^ ameliorates health,^21,22,23^ and reduces disparities in coverage, access, and health outcomes by race, ethnicity, and insurance coverage status in individuals younger than 65 years.^18,19,21,23^ In one study, the association of Medicare eligibility with health and access was also assessed among transgender and cisgender adults, finding that Medicare eligibility may lead to improvements in health and access among both groups, with effects of greater magnitude among transgender individuals.^24^ However, to our knowledge, no studies have evaluated the effect of Medicare eligibility on LGBTQI+ populations more broadly, including sexual minority adults. Partly due to limitations in SOGI data collection,^4,8,16,25,26,27^ the gaps in knowledge regarding the benefits of becoming Medicare-eligible to older adults who identify as LGBTQI+ encompass questions around how Medicare eligibility impacts their use of health services, forms of coverage, and aging.^8,9,28,29,30^ These knowledge gaps may contribute to the failure to consider the needs of older adults who identify as LGBTQI+ in policies and social services. The absence of LGBTQI+ competence in policies and social services geared toward older adults is especially concerning as the generation most impacted by the HIV/AIDS epidemic advances into old age.^8,25,31^

In this study, we used a regression discontinuity design (RDD) to estimate the association of Medicare eligibility at age 65 years with health insurance coverage, access to care, and health status by sexual orientation and by gender identity. We analyzed the natural experiment that occurs at age 65 years when nearly all US citizens become Medicare eligible. Our approach compares outcomes for individuals who are younger and older than the 65-year eligibility threshold to ensure that the observable and unobservable characteristics of individuals were similar and that only their eligibility for Medicare differed. RDD is an increasingly common approach to estimate plausibly causal effects in clinical,^32^ policy,^20,21,33,34^ and economics research.^18,22,35^

## Methods

This study was exempted from review by the Yale University Institutional Review Board because it was not human participant research. We followed the Strengthening the Reporting of Observational Studies in Epidemiology (STROBE) reporting guidelines.

### Study Design and Population

Our RDD assessed the association between becoming 65 years old—when nearly all US adults become eligible for Medicare—and insurance coverage, access to care, and self-reported health status. The key assumption is that there is a smooth and continuous relationship between age and the other determinants of the selected outcomes around the discontinuity—ie, that there are not discontinuous changes in other predictors of the outcomes during a narrow window at approximately 65 years old. We demonstrated empirically that this assumption was not violated in our study setting—a finding that is consistent with numerous other studies of this age-based discontinuity.^32,35^ Then, following the literature,^36^ we estimated RDD models without individual-level predictors beyond age in years (ie, the running variable) as these observed predictors have been shown to be balanced around the discontinuity.

We analyzed 2014 to 2021 data from the Behavioral Risk Factor Surveillance System (BRFSS), a nationwide survey of US household information on health risks, behaviors, and care, administered and maintained by the US Centers for Disease Control and Prevention. The BRFSS is the largest annual health survey, with a median state-level combined (telephone landline and cell phone) response rate (range) during our study period of 47.2% (44.0%-49.9%). Beginning in 2014, new demographic questions were implemented by the BRFSS to record respondents’ sexual orientation and gender identity, leaving states the option to collect this information. In this study, we referred to respondents who identified as a sexual minority (lesbian, gay, bisexual, or “other/something else”) as LGB+, with the plus symbol (+) to include persons of another sexual minority identity, and to respondents who identified as “straight, that is, not gay,” as heterosexual. Regarding gender identity, we referred to those who identified as transgender or gender nonconforming as transgender or gender diverse (TGD), and all others, as cisgender. Consistent with existing literature, we excluded individuals who responded, “don’t know,” “not sure,” or “refused” to both the sexual orientation and gender identity questions.^1,2,37^ Respondents who provided sexual orientation but not gender identity or vice versa were included (eMethods and eTable 1 in Supplement 1 provide additional details on exclusions and missing data). We limited our primary sample to adults aged 51 to 79 years in 43 US states, excluding 7 states and the District of Columbia because they did not report SOGI data in the BRFSS for any of the study years (eMethods in Supplement 1).

### Study Variables

Consistent with prior literature,^21^ we assessed outcomes in 3 domains: health insurance coverage, access to health care services, and self-reported health status. First, we assessed whether respondents had any form of health insurance coverage. Second, we assessed health care access using several measures: (1) whether respondents had “a usual source of care”; (2) whether respondents “did not see a doctor due to cost in the past 12 months”; and (3) whether respondents received an influenza vaccine in the past 12 months. Although there are a variety of factors that affect influenza vaccination uptake,^38,39,40,41,42,43,44,45^ receipt of an influenza vaccine is consistent with previous research as a proxy for access to care.^21,46^ Given that respondents reported cost barriers to care and receipt of the influenza vaccine during the past 12 months, as opposed to the time of interview, we analyzed these 2 outcomes using a donut RDD that excluded observations at age 65 years, a common approach in the literature.^47^ Third, we assessed self-reported health status. For a primary outcome, we assessed whether respondents reported being in good, very good, or excellent health, which we referred to as good or better health. As secondary outcomes, we assessed whether respondents reported being in fair or poor health. We assessed self-reported individual characteristics from the BRFSS data, including sex, gender identity, sexual orientation, marital status, employment status, education level, income level, and race and ethnicity.

### Statistical Analysis

Using our RDD, we estimated the adjusted discontinuity for each outcome using local linear regression with a uniform kernel, allowing age trends to differ above and below the discontinuity. The vertical distance at age 65 years between the local regression lines fit above and below the threshold measured the adjusted discontinuity (the estimate of interest). In the primary analyses, we used a data-driven method that traded off bias and variance to automatically select an optimal bandwidth—the age range around the cutoff used to fit local linear regressions—and account for discreteness in the running variable (ie, that age is measured in years). Because a discrete running variable requires additional extrapolation of the local linear regression models to the 65-years-old threshold, we reported bias-adjusted confidence intervals that account for sampling error and the additional uncertainty due to potential misspecification.^36,48^ We stratified by SOGI status and documented how estimates differed between individuals who identified as LGB+ versus heterosexual (hereafter, LGB+ individuals and heterosexual individuals) and between those who identified as TGD versus cisgender (hereafter, TGD individuals and cisgender individuals). To tractably compute differences-in-discontinuities by sexual orientation status, we used a linear regression model with interactions (eMethods in Supplement 1). We included calculations for TGD versus cisgender individuals in Supplement 1 because of smaller sample sizes.

We conducted several sensitivity analyses and placebo tests used frequently in the literature.^21,49^ First, we evaluated the robustness of the study results to alterations in the statistical model for RDD.^21^ Second, we repeated analyses using survey weights. Although this reduced our precision, we could assess whether the results were qualitatively similar to our main findings. Third, in placebo tests, we tested for covariate smoothness—ie, whether there were discontinuities in respondent characteristics at age 65 years—and for discontinuities in the number of respondents at age 65 years.^49^

In secondary analyses, for the primary outcomes we assessed the mean state-level disparity between LGB+ and heterosexual adults aged 51 to 64 years (nearly Medicare eligible; eMethods in Supplement 1). We ranked states according to the mean of the disparities among these nearly eligible LGB+ individuals compared with their heterosexual counterparts across the 4 outcomes. We conducted subgroup analyses among the top-10 high-disparity states, and an alternative definition of this group of states to test the robustness of our state-level results among high-disparity states. Additionally, we assessed the associations of Medicare eligibility separately by US Census region to explore geographic heterogeneity. Lastly, we conducted 2 separate subgroup analyses in which we (1) limited the study population to married individuals, and (2) limited the study period to 2016 to 2021—the period after which the *Obergefell v Hodges* Supreme Court decision legalized same-sex marriage nationwide.^50^

The statistical analyses were performed using Stata, version 17 (StataCorp LLC) and the RDHonest (Honest Inference in Regression Discontinuity Designs) package^48,51^ from September 2022 through April 2023. We used 2-tailed tests and defined statistical significance as α = .05.

## Results

### Study Population

The study population consisted of 927 952 respondents (mean [SD] age, 64.4 [7.7] years; 524 972 [56.6%] females and 402 670 [43.4%] males), of whom, 28 077 respondents (3.03%) identified as LGB+ and 3286 (0.35%) as TGD (eTable 2 in Supplement 1 for weighted estimates of all descriptive characteristics and eTable 3, stratification by sexual orientation). Demographic characteristics were similar for just younger than Medicare eligibility age compared with those just older than Medicare eligibility age (Table 1); however, we did find some difference in employment rates at age 65 years. Although race and ethnicity data were collected and are reported, due to small sample size and statistical power limitations, further analyses considering intersectionality of more than 1 minority identity (eg, race or ethnicity and sexual orientation or gender identity) could not be completed.

**Table 1.** Characteristics of the Study Population and Medicare Age-Related Discontinuities

Characteristic	Sample means, No. (%)^a^		Change at age 65 y	
	Age 51-64 y	Age 65-79 y	Expected mean, %^b^	Adjusted discontinuity (95% CI)^c^
Sex				
Female	260 020 (55.4)	264 952 (57.8)	55.97	−0.20 (−0.84 to 0.44)
Male	209 400 (44.6)	193 270 (42.2)	44.00	0.19 (−0.45 to 0.84)
“Don’t know/not sure/refused”	160 (0)	150 (0)	0.04	0 (−0.02 to 0.02)
Gender identity				
Cisgender	465 905 (99.3)	454 272 (99.2)	99.30	0.01 (−0.08 to 0.11)
Transgender or gender diverse	1797 (0.4)	1489 (0.3)	0.37	−0.04 (−0.09 to 0)
“Don’t know/not sure/refused”	1593 (0.3)	2209 (0.5)	0.32	0.04 (−0.03 to 0.11)
Sexual orientation				
Heterosexual	444 871 (95.0)	435 419 (95.2)	95.24	0.19 (−0.12 to 0.49)
LGB+	16 129 (3.4)	11 948 (2.6)	2.96	−0.13 (−0.29 to 0.03)
“Don’t know/not sure/refused”	7419 (1.6)	9882 (2.2)	1.84	−0.03 (−0.24 to 0.18)
Marital status				
Married	281 687 (60.4)	250 259 (54.9)	59.56	−0.13 (−0.82 to 0.56)
Member of unmarried couple	10 213 (2.2)	5202 (1.1)	1.55	−0.02 (−0.13 to 0.09)
Divorced, widowed, separated	125 381 (26.9)	172 821 (37.9)	30.54	−0.39 (−0.87 to 0.08)
Never married	49 108 (10.5)	27 676 (6.1)	8.41	−0.03 (−0.45 to 0.39)
Employed	290 533 (62.4)	89 738 (19.7)	38.89	−3.22 (−4.69 to −1.76)
Education level				
<High school	29 602 (6.3)	29 135 (6.4)	5.09	0.28 (−0.16 to 0.71)
High school graduate	126 789 (27.1)	123 214 (27.0)	25.45	0.28 (−0.64 to 1.20)
Some college	129 602 (27.7)	126 522 (27.7)	29.10	−0.09 (−0.59 to 0.40)
College graduate	182 176 (38.9)	178 288 (39.0)	40.20	−0.04 (−1.11 to 1.02)
Income level, $				
<10 000	19 210 (4.8)	10 791 (2.9)	3.29	0.26 (−0.15 to 0.67)
10 000-24 999	70 605 (17.4)	81 525 (21.9)	20.21	−1.03 (−1.41 to −0.65)
25 000-49 999	83 011 (20.5)	111 687 (30.0)	26.00	0.30 (−0.60 to 1.20)
50 000-74 999	66 745 (16.5)	66 579 (17.9)	18.12	0.29 (−0.25 to 0.83)
≥75 000	165 212 (40.8)	101 275 (27.2)	31.71	−0.07 (−0.92 to 0.77)
Race and ethnicity				
Black	37 331 (8.1)	29 468 (6.5)	7.25	0.37 (−0.06 to 0.80)
Hispanic	24 629 (5.3)	13 672 (3.0)	3.55	0.06 (−0.21 to 0.33)
White	369 710 (80.2)	384 184 (85.3)	84.15	−0.85 (−1.45 to −0.26)
Other^d^	29 554 (6.4)	23 090 (5.1)	5.14	0.43 (0.08 to 0.79)

Abbreviation: LGB+, lesbian, gay, bisexual, or another sexual minority identity.

^a^ Raw counts of respondents and unweighted proportions in percentages.

^b^ Expected mean at age 65 years based on local linear association between age and the outcome in each row. The expected means contain the counterfactual outcome at age 65 years in the absence of treatment (the expected outcome at 65 years without Medicare).

^c^ Adjusted discontinuity estimates are in percentage points and report the RDHonest estimates and bias-adjusted confidence intervals (refer to the Methods section for more detail on the statistical model).

^d^ Included American Indian or Alaskan Native, Asian, Native Hawaiian or other Pacific Islander, “other race,” and “multiracial.”

### Changes Before and After Age 65 Years

**Figure 1.**
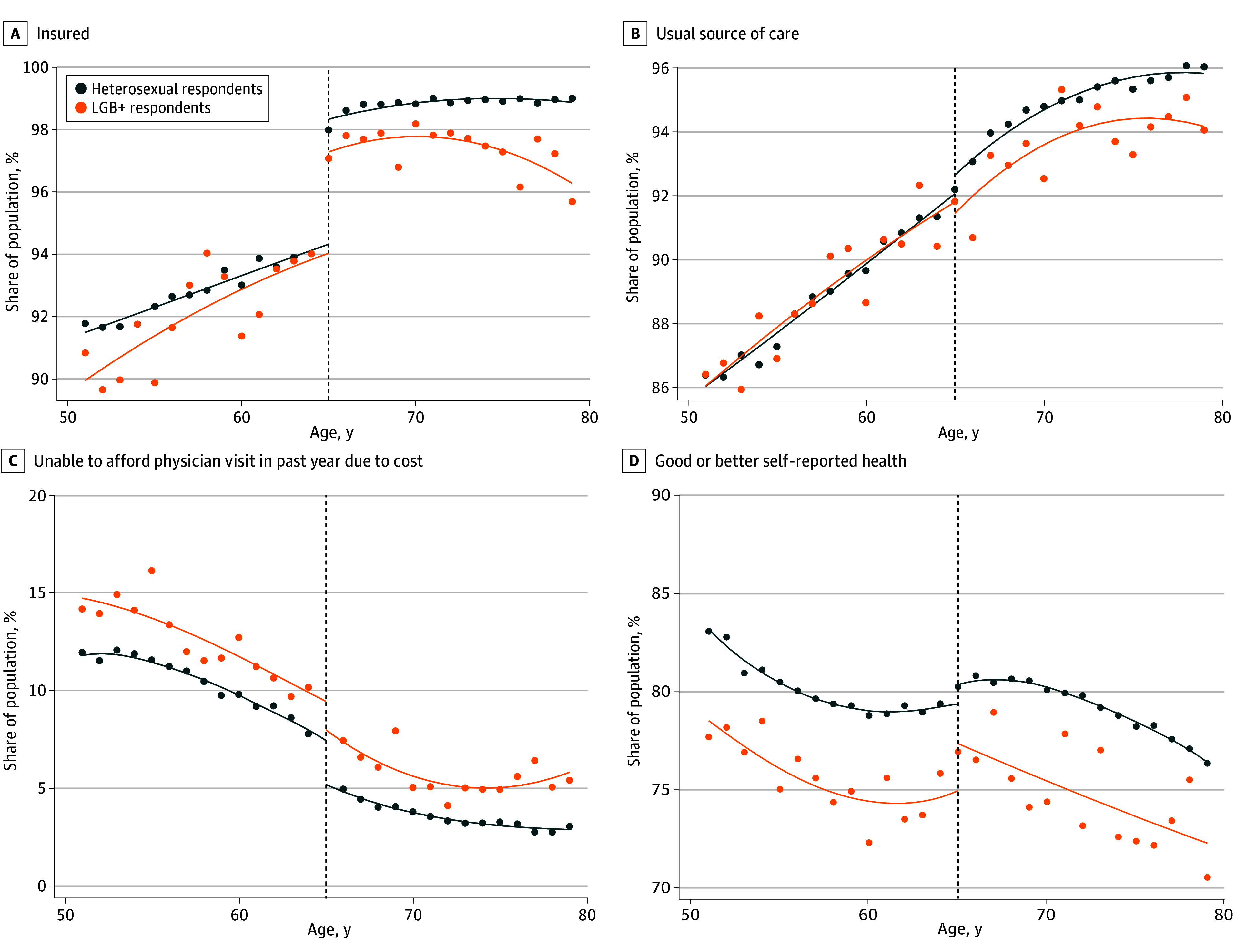
Medicare Eligibility Age-Related Discontinuities in Insurance Coverage, Access to Care, and Self-Reported Health Status, by Sexual Orientation at the National Level Each panel plots the share of the study population reporting each outcome by age in years, separately for heterosexual and LGB+ respondents, during the study period (2014-2021). For illustrative purposes, the line of best fit is based on a local regression model using the optimal bandwidth selected by the RDHonest model for each outcome separately for heterosexual and LGB+ respondents (refer to the eMethods in Supplement 1). The Medicare eligibility age threshold at 65 years is represented by the dashed black line. LGB+ indicates lesbian, gay, bisexual, or another sexual minority identity.

Medicare eligibility at age 65 years was associated with a 4.2−percentage point (pp) increase in health insurance coverage. This increase was greater in magnitude for heterosexual individuals (from 94.4% to 98.5% [4.2 pp; 95% CI, 4.0-4.4 pp]) compared with LGB+ individuals (from 94.1% to 97.7% [3.6 pp; 95% CI, 2.3-4.8 pp]) (Table 2 and Figure 1).

**Table 2.** Medicare Eligibility Age-Related Discontinuities in Insurance Coverage, Access to Care, and Self-Reported Health Status, by Sexual Orientation at the National Level^a^

Study group	Sample means, %		Change at age 65 y	
	Age 51-64 y	Age 65-79 y	Expected mean, %^b^	Adjusted discontinuity (95% CI)^c^
**Full sample**				
Insurance coverage	92.7	98.7	94.3	4.15 (3.96 to 4.35)
Health care access				
Have a usual source of care	88.8	94.5	92.0	1.10 (0.87 to 1.33)
Unable to see physician in past year due to cost	10.5	3.9	7.7	−2.32 (−2.87 to −1.77)
Received an influenza vaccination in past year	46.3	62.6	54.3	2.23 (1.10 to 3.36)
Self-reported health				
Poor	6.2	5.8	5.9	−0.29 (−0.76 to 0.18)
Fair	14.2	15.0	15.1	−0.84 (−1.39 to −0.30)
Good or better	79.6	79.1	79.0	1.17 (0.39 to 1.96)
**Heterosexual individuals**				
Insurance coverage	92.9	98.8	94.4	4.17 (3.98 to 4.36)
Health care access				
Have a usual source of care	89.0	94.6	92.0	1.14 (0.90 to 1.39)
Unable to see physician in past year due to cost	10.3	3.7	7.5	−2.32 (−2.87 to −1.76)
Received an influenza vaccination in past year	46.2	62.8	54.4	2.23 (1.07 to 3.39)
Self-reported health				
Poor	6.1	5.7	5.8	−0.24 (−0.71 to 0.23)
Fair	13.9	14.8	14.9	−0.81 (−1.36 to −0.27)
Good or better	80.0	79.5	79.3	1.09 (0.30 to 1.89)
**LGB+ individuals**				
Insurance coverage	92.1	97.5	94.1	3.55 (2.31 to 4.80)
Health care access				
Have a usual source of care	88.9	93.4	92.0	0.02 (−1.52 to 1.55)
Unable to see physician in past year due to cost	12.6	5.8	9.8	−1.99 (−4.05 to 0.07)
Received an influenza vaccination in past year	51.7	61.7	58.0	1.10 (−1.59 to 3.78)
Self-reported health				
Poor	7.4	7.8	7.3	−0.20 (−2.14 to 1.74)
Fair	17.0	17.0	18.8	−2.81 (−4.82 to −0.80)
Good or better	75.6	75.2	74.8	2.60 (−0.40 to 5.60)

Abbreviation: LGB+, lesbian, gay, bisexual, or another sexual minority identity.

^a^ All estimates are unweighted.

^b^ Expected mean at age 65 years based on the local linear association between age and the outcome in each row. The expected means contain the counterfactual outcome at age 65 years in the absence of treatment (the expected outcome at 65 years without Medicare).

^c^ Adjusted discontinuity estimates are in percentage points and report the RDHonest estimates and bias-adjusted confidence intervals (refer to the Methods section for more detail on the statistical model).

Access to care improved at age 65 years for our overall study population (Table 2). However, when we stratified by sexual orientation, point estimates of the improvements in all 3 access measures at age 65 years were larger for heterosexual respondents compared with LGB+ respondents, although the confidence intervals overlapped (Table 2 and Figure 1). The share of respondents with a usual source of care had a greater increase among heterosexual individuals (from 92.0% to 93.2% [1.1 pp; 95% CI, 0.9-1.4 pp]) compared with LGB+ individuals (from 92.0% to 92.0% [0 pp; 95% CI, −1.5 to 1.6 pp]), with the latter estimate having less precision due to the smaller sample size of the LGB+ respondents. The reduction in the share of respondents unable “to see a doctor due to cost” was greater in magnitude for heterosexual individuals (from 7.5% to 5.2% [ −2.3 pp; 95% CI, −2.9 to −1.8 pp]) compared with LGB+ individuals (from 9.8% to 7.8% [−2.0 pp; 95% CI, −4.1 to 0.1 pp]). Finally, the share of respondents that reported getting an influenza vaccination in the past year increased more heterosexual individuals (from 54.4% to 56.6% [2.2 pp; 95% CI, 1.1 to 3.4 pp]) than LGB+ individuals (from 58.0% to 59.1% [1.1 pp; 95% CI, −1.6 to 3.8 pp]), although the confidence intervals were overlapping (eFigure 1 in Supplement 1).

For self-reported health, we observed a discontinuous increase in the share of respondents reporting good or better health at age 65 years. The effect was larger for LGB+ (from 74.8% to 77.4% [2.6 pp; 95% CI, −0.4 to 5.6 pp]) than for heterosexual respondents (from 79.3% to 80.4% [1.1 pp; 95% CI, 0.3 to 1.9 pp]), although point estimates for those identifying as LGB+ were less precise. We lacked the statistical power to precisely estimate adjusted discontinuities in disparities (eTable 4 in Supplement 1).

### State-Level Disparities by Sexual Orientation

**Figure 2.**
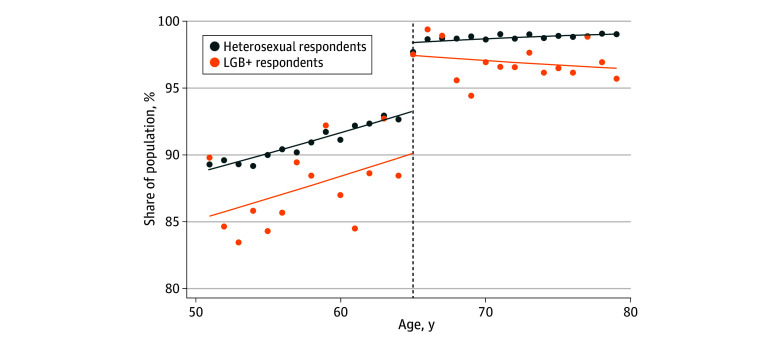
Medicare Eligibility Age-Related Discontinuity in Insurance Coverage, by Sexual Orientation in the Top-10 High-Disparity States The share of the study population in the top-10 high-disparity states that report having any health insurance coverage during the study period (2014-2021) by age in years is plotted separately for heterosexual and LGB+ respondents. The top-10 high-disparity states based on the mean disparity among the nearly eligible (age 51-64 years) were Mississippi, Tennessee, Kansas, Texas, Missouri, Oklahoma, Pennsylvania, New Jersey, Iowa, and New Mexico. For illustrative purposes, the line of best fit is based on a local regression model using the optimal bandwidth selected by the RDHonest model for each outcome separately for heterosexual and LGB+ respondents (refer to eMethods in Supplement 1). The Medicare eligibility age threshold of 65 years is represented by the dashed black line. LGB+ indicates lesbian, gay, bisexual, or another sexual minority identity.

The magnitude of disparities among the nearly eligible for Medicare differed by state (eTables 5 through 8 in Supplement 1). For example, Mississippi had the largest coverage disparity, with coverage rates 8.8 pp higher among heterosexual compared to LGB+ respondents (eTable 9 in Supplement 1). The 10 states with the largest disparities between heterosexual and LGB+ respondents 51 to 64 years old were Mississippi, Tennessee, Kansas, Texas, Missouri, Oklahoma, Pennsylvania, New Jersey, Iowa, and New Mexico (eTable 9 in Supplement 1). Among these high-disparity states, Medicare eligibility at age 65 years was associated with larger improvements in coverage, access to a usual source of care, and self-reported health for LGB+ than for heterosexual respondents (Figure 2 and Table 3; eFigure 2 in Supplement 1), although confidence intervals were overlapping. These results were qualitatively similar for our alternative definition of high-disparity states (eTables 10 and 11 in Supplement 1).

**Table 3.** Medicare Eligibility Age-Related Discontinuities in Insurance Coverage, Access to Care, and Self-Reported Health Status, by Sexual Orientation in High-Disparity States^a^

Study group	Sample means, %		Change at age 65 y	
	Age 51-64 y	Age 65-79 y	Expected mean, %^b^	Adjusted discontinuity (95% CI)^c^
**Full sample**				
Insurance coverage	90.7	98.6	93.0	5.08 (4.45 to 5.70)
Health care access				
Have a usual source of care	88.6	94.6	92.1	0.59 (−0.14 to 1.33)
Unable to see physician in past year due to cost	11.8	3.9	8.3	−2.70 (−3.85 to −1.55)
Received an influenza vaccination in past year	47.2	64.0	57.0	−0.02 (−1.99 to 1.94)
Self-reported health				
Poor	6.8	6.4	6.6	−0.77 (−1.61 to 0.07)
Fair	15.4	16.5	16.8	−1.21 (−2.08 to −0.33)
Good or better	77.9	77.2	76.6	1.81 (0.47 to 3.15)
**Heterosexual individuals**				
Insurance coverage	91.1	98.7	93.3	4.96 (4.32 to 5.59)
Health care access				
Have a usual source of care	88.9	94.7	92.3	0.59 (−0.19 to 1.38)
Unable to see physician in past year due to cost	11.5	3.7	8.1	−2.83 (−3.98 to −1.67)
Received an influenza vaccination in past year	47.2	64.3	57.1	0.14 (−1.82 to 2.11)
Self-reported health				
Poor	6.6	6.1	6.5	−0.80 (−1.63 to 0.04)
Fair	15.0	16.2	16.3	−0.99 (−1.92 to −0.06)
Good or better	78.5	77.7	77.3	1.63 (0.27 to 2.98)
**LGB+ individuals**				
Insurance coverage	87.6	97.0	90.8	6.73 (3.25 to 10.22)
Health care access				
Have a usual source of care	86.8	93.4	89.6	1.36 (−3.31 to 6.03)
Unable to see physician in past year due to cost	16.0	6.2	13.5	−1.89 (−8.03 to 4.26)
Received an influenza vaccination in past year	52.4	61.7	61.8	−0.67 (−11.55 to 10.21)
Self-reported health				
Poor	8.6	8.7	7.7	0.88 (−4.09 to 5.84)
Fair	21.7	19.3	24.1	−5.91 (−11.47 to −0.36)
Good or better	69.8	72.0	67.6	5.53 (−0.94 to 11.99)

Abbreviation: LGB+, lesbian, gay, bisexual, or another sexual minority identity.

^a^ All estimates are unweighted.

^b^ Expected mean at age 65 years based on the local linear association between age and the outcome in each row. The expected means contain the counterfactual outcome at age 65 years in the absence of treatment (the expected outcome at 65 years without Medicare).

^c^ Adjusted discontinuity estimates are in percentage points and report the RDHonest estimates and bias-adjusted confidence intervals (refer to Methods sections for more detail on the statistical model).

### Supplemental and Sensitivity Analyses

Among married respondents, increases in coverage were greater for LGB+ individuals than for heterosexual individuals, although the confidence intervals were overlapping, with the other results being qualitatively similar to those of the primary analyses (eTable 12 in Supplement 1). Subgroup analyses that were limited to the post-Obergefell period were qualitatively similar to our primary results (eTable 13 in Supplement 1). When we stratified by US Census region, results were variable with outcomes in the Northeast region being qualitatively similar to those of the primary analysis at the national level and outcomes in the Midwest region being most qualitatively similar to those of the analysis among the top-10 high-disparity states, with the exception of absent improvement in self-reported health (eFigures 3 through 6 in Supplement 1). Stratifying by gender identity revealed inequities between nearly eligible cisgender and TGD respondents, which were more pronounced than those by sexual orientation in our primary analysis; these inequities also remained largely unchanged after Medicare eligibility (eFigure 7 and eTable 14 in Supplement 1).

Sensitivity analyses, including using alternative kernels, different bounds on the second derivative of age function, and weights in our RDD model supported our primary conclusions (eTables 15 through 17 in Supplement 1). As for placebo tests, except for employment and individuals of other races, we did not find any discontinuous changes at age 65 years in respondent characteristics (Table 1; eFigure 8 in Supplement 1) nor any discontinuous changes in the number of respondents (eFigures 9 through 11 in Supplement 1).

## Discussion

As evidenced by our comparison of individuals younger and older than 65, Medicare eligibility was associated with overall increases in insurance coverage rates, access to care, and self-reported health—consistent with prior evidence.^18,19,20,21,22,23^ Stratifying by sexual orientation, we found that eligibility for Medicare coverage was often associated with improvements in coverage and access to care that were larger in magnitude for heterosexual adults rather than LGB+ adults, even when LGB+ adults had similar or lower coverage rates and access to care before age 65 years. For self-reported health, we found a larger improvement among LGB+ individuals at age 65 years compared with heterosexual respondents. However, when limited to married individuals, the magnitude of improvement in insurance coverage became greater for LGB+ than for heterosexual respondents, and the strength of the larger improvement in health among LGB+ individuals became stronger. This finding suggests that marriage plays a role in health insurance coverage and health status for LGB+ adults, which is consistent with previous research.^15,52,53,54^

In contrast to prior evidence showing that Medicare eligibility was associated with broad reductions in racial and ethnic disparities,^18,21^ we did not find consistent evidence that gaining Medicare eligibility at age 65 years reduced LGBTQI+ disparities. One key mechanism by which Medicare was found to be associated with reduced racial and ethnic disparities was by closing coverage gaps between nearly eligible populations. However, nearly Medicare eligible coverage rates at the national level were similar between the LGB+ and heterosexual respondents in our study findings despite access to care and health generally being poorer for LGB+ respondents. In contrast, self-reported health status gains were greater for LGB+ than heterosexual individuals, suggesting that LGB+ adults are more sensitive in health to Medicare eligibility, which is consistent with similar literature demonstrating greater improvement in health among transgender than cisgender adults after becoming Medicare-eligible. These findings complement prior research on the broad determinants of LGBTQI+ disparities, including structural stigma and discrimination; violence and victimization; deferral of care due to cost barriers or poor clinician competence regarding LGBTQI+ lived experiences and health; and higher levels of poverty among some LGBTQI+ populations.^3,4,8,25,29,30,31,37,55,56^ It is most likely that Medicare eligibility is associated with improvements of a larger magnitude for adults who identify as heterosexual rather than LGB+, with the exception of health status, because the largest disparity by sexual orientation in our study for individuals younger than 65 years was in health status and Medicare eligibility alone may be insufficient to overcome structural and interpersonal forms of discrimination (eg, prior employment discrimination, differences in supplemental coverage, clinicians who are not competent in providing LGBTQI+-affirming health care, cost barriers to care). Additional interventions are likely necessary to create larger improvements in insurance coverage and access to care for LGB+ beneficiaries. Future research is needed to assess differences in how LGB+ and heterosexual beneficiaries navigate the Medicare program, and to what extent prior employment discrimination, differences in supplemental coverage, and/or the cost burden of health care interact with Medicare eligibility.^6,57^ The persistence of disparities within Medicare-eligible populations also suggests improving clinician knowledge of LGBTQI+ health could serve as a lever for reducing the disparities that we observed while waiting for the broader structural impediments to be addressed.

Our study also uncovered substantial variation across states and US Census regions at the level of LGB+ disparities—a focal question raised in the first-ever *Federal Evidence Agenda on LGBTQI+ Equity*^16^—and found that Medicare eligibility was associated with improved health insurance coverage and access to a usual source of care that were greater in magnitude for LGB+ respondents rather than for heterosexual respondents in high-disparity states, the opposite pattern to our national-level results. Consistent with previous research,^58^ states with the largest disparities (eg, Mississippi) tended to be in the Southern and Central regions, which have greater documented hostility toward sexual and gender minority populations and fewer legal protections for them.^59,60^ Unwarranted state-level variation in policies and disparities highlights the important role of US federal policies—eg, the Equality Act^61^ that would enact universal legal protections for sexual and gender minority populations, regardless of state of residence—to advance health equity in areas of the US where disparities may be tied to harmful state or local policies.^60^

### Limitations

Our study had several limitations. First, our running variable age was only available in discrete years, complicating extrapolation around the discontinuity. To address this issue, we used the RDHonest package,^51^ developed specifically for discrete RDD to report conservative, bias-adjusted confidence intervals that address the uncertainty due to the additional extrapolation needed. Second, RDD estimates the short-run impact of Medicare eligibility and may not capture the association between Medicare eligibility and outcomes that respond with a lag period. Third, our results may not generalize to other measures of insurance coverage, access to care, and health status that were not included in our study. Fourth, it is possible that changes in other predictors of our outcomes in individuals younger than 65 years versus older than 65 years (besides Medicare eligibility) could bias our results. We present evidence of small and generally statistically insignificant changes in study characteristics at age 65 years, but other changes around age 65 years (eg, retirement) may have contributed to our results. Fifth, statistical power was limited by the small sample sizes of LGB+ and TGD individuals. Hence, we cautiously interpreted our results, in line with a previous study that assessed how Medicare eligibility affected access to care and health outcomes among individuals who identify as transgender,^24^ and led with unweighted estimates for precision (weighted results were less precise but qualitatively similar). The small sample sizes, even in a large survey such as the BRFSS, highlight the importance of improving SOGI data collection,^4,16^ including by oversampling LGB+ and TGD respondents and requiring SOGI questions in the core survey questionnaire of the BRFSS.^27,37^ Although inclusion of SOGI questions is not frequently associated with survey incompletion and rates of SOGI item nonresponse are low, establishing trust with survey respondents, which may be facilitated by ensuring privacy and confidentiality, should be prioritized.^4,16,62,63^

## Conclusions

The findings of this cross-sectional study suggest that expanding Medicare is insufficient to advancing health equity for sexual and gender minority populations. However, Medicare eligibility may be able to partially close gaps in coverage and access to care for sexual minority individuals in the highest-disparity states. Our findings highlight the possible scope for US federal policy that promote health equity in places where disparities may be tied to harmful state or local policies. These findings also underscore that an expansion of Medicare coverage may be just a first step toward advancing health equity for LGBTQI+ populations, who experience health inequities driven by a broad set of structural factors.

## Notes

Supplement 1. eMethods. Supplemental Description of Methods eFigure 1. Medicare eligibility age-related discontinuities in access and self-reported health by sexual orientation eFigure 2. Medicare eligibility age-related discontinuities in access and self-reported health by sexual orientation in the top ten “high disparity” states eFigure 3. Medicare eligibility age-related discontinuities in coverage, access and self-reported health by sexual orientation in the United States Northeast eFigure 4. Medicare eligibility age-related discontinuities in coverage, access and self-reported health by sexual orientation in the United States Midwest eFigure 5. Medicare eligibility age-related discontinuities in coverage, access and self-reported health by sexual orientation in the United States South eFigure 6. Medicare eligibility age-related discontinuities in coverage, access and self-reported health by sexual orientation in the United States West eFigure 7. Medicare eligibility age-related discontinuities in coverage, access, and self-reported health by gender identity eFigure 8. Covariate smoothness test for select study population characteristics eFigure 9. Smoothness tests for compositional change in reported sexual orientation and gender identity eFigure 10. Smoothness tests for the number of respondents by sexual orientation eFigure 11. Smoothness tests for the number of respondents by gender identity eTable 1. Missingness in primary outcomes eTable 2. Weighted study pop. characteristics and Medicare age-related discontinuities eTable 3. Demographic characteristics of the study population by sexual orientation eTable 4. Difference-in-discontinuities in study outcomes at the national level and among “high disparity” states eTable 5. Differences by sexual orientation in share insured for 51-64 year-olds by state eTable 6. Differences by sexual orientation in usual source of care for 51-64 year-olds by state eTable 7. Differences by sexual orientation in cost barriers for 51-64 year-olds by state eTable 8. Differences by sexual orientation in good or better health for 51-64 year-olds by state eTable 9. Mean disparities in the top ten “high disparity” states between heterosexual and LGB+ respondents, ages 51-64 eTable 10. Mean disparities in the top ten “high disparity” states between heterosexual and LGB+ respondents, ages 51-55 eTable 11. Medicare eligibility age-related discontinuities in coverage, access, and self-reported health by sexual orientation in “high disparity” states, based on respondents aged 51-55 eTable 12. Medicare eligibility age-related discontinuities in coverage, access, and self-reported health among married individuals by sexual orientation at the national level eTable 13. Medicare eligibility age-related discontinuities in coverage, access, and self-reported health by sexual orientation in the period post-Obergefell v. Hodges at the national level eTable 14. Medicare eligibility age-related discontinuities in coverage, access, and self-reported health by gender identity eTable 15. Medicare eligibility age-related discontinuities in coverage, access, and self-reported health by sexual orientation, weighted eTable 16. Medicare eligibility age-related discontinuities in coverage, access, and self-reported health by gender identity, weighted eTable 17. Robustness of Medicare eligibility age-related discontinuities to alterations in the statistical model

Supplement 2. Data Sharing Statement
