# Supplementary material for: Medicare Eligibility and Changes in Coverage, Access to Care, and Health by Sexual Orientation and Gender Identity
Source: JAMA Health Forum. 2024 Jul 5;5(7):e241756. doi: 10.1001/jamahealthforum.2024.1756 (PMC11227074; doi:10.1001/jamahealthforum.2024.1756)
Supplement: Supplement 1. — eMethods. Supplemental Description of Methods eFigure 1. Medicare eligibility age-related discontinuities in access and self-reported health by sexual orientation eFigure 2. Medicare eligibility age-related discontinuities in access and self-reported health by sexual orientation in the top ten “high disparity” states eFigure 3. Medicare eligibility age-related discontinuities in coverage, access and self-reported health by sexual orientation in the United States Northeast eFigure 4. Medicare eligibility age-related discontinuities in coverage, access and self-reported health by sexual orientation in the United States Midwest eFigure 5. Medicare eligibility age-related discontinuities in coverage, access and self-reported health by sexual orientation in the United States South eFigure 6. Medicare eligibility age-related discontinuities in coverage, access and self-reported health by sexual orientation in the United States West eFigure 7. Medicare eligibility age-related discontinuities in coverage, access, and self-reported health by gender identity eFigure 8. Covariate smoothness test for select study population characteristics eFigure 9. Smoothness tests for compositional change in reported sexual orientation and gender identity eFigure 10. Smoothness tests for the number of respondents by sexual orientation eFigure 11. Smoothness tests for the number of respondents by gender identity eTable 1. Missingness in primary outcomes eTable 2. Weighted study pop. characteristics and Medicare age-related discontinuities eTable 3. Demographic characteristics of the study population by sexual orientation eTable 4. Difference-in-discontinuities in study outcomes at the national level and among “high disparity” states eTable 5. Differences by sexual orientation in share insured for 51-64 year-olds by state eTable 6. Differences by sexual orientation in usual source of care for 51-64 year-olds by state eTable 7. Differences by sexual orientation in cost barriers for 51-64 year-o [file jamahealthforum-e241756-s001.pdf]

# Supplemental Online Content

Gavulic KA, Wallace J. Medicare eligibility and changes in coverage, access to care, and health by sexual orientation and gender identity. *JAMA Health Forum.* Published online July 5, 2024. doi:10.1001/jamahealthforum.2024.1756

**eMethods.** Supplemental Description of Methods

**eFigure 1.** Medicare eligibility age-related discontinuities in access and self-reported health by sexual orientation

**eFigure 2.** Medicare eligibility age-related discontinuities in access and self-reported health by sexual orientation in the top ten “high disparity” states

**eFigure 3.** Medicare eligibility age-related discontinuities in coverage, access and self-reported health by sexual orientation in the United States Northeast

**eFigure 4.** Medicare eligibility age-related discontinuities in coverage, access and self-reported health by sexual orientation in the United States Midwest

**eFigure 5.** Medicare eligibility age-related discontinuities in coverage, access and self-reported health by sexual orientation in the United States South

**eFigure 6.** Medicare eligibility age-related discontinuities in coverage, access and self-reported health by sexual orientation in the United States West

**eFigure 7.** Medicare eligibility age-related discontinuities in coverage, access, and self-reported health by gender identity

**eFigure 8.** Covariate smoothness test for select study population characteristics

**eFigure 9.** Smoothness tests for compositional change in reported sexual orientation and gender identity

**eFigure 10.** Smoothness tests for the number of respondents by sexual orientation

**eFigure 11.** Smoothness tests for the number of respondents by gender identity

**eTable 1.** Missingness in primary outcomes

**eTable 2.** Weighted study pop. characteristics and Medicare age-related discontinuities

**eTable 3.** Demographic characteristics of the study population by sexual orientation

**eTable 4.** Difference-in-discontinuities in study outcomes at the national level and among “high disparity” states

© 2024 Gavulic KA et al. *JAMA Health Forum.*

**eTable 5.** Differences by sexual orientation in share insured for 51-64 year-olds by state

**eTable 6.** Differences by sexual orientation in usual source of care for 51-64 year-olds by state

**eTable 7.** Differences by sexual orientation in cost barriers for 51-64 year-olds by state

**eTable 8.** Differences by sexual orientation in good or better health for 51-64 year-olds by state

**eTable 9.** Mean disparities in the top ten "high disparity" states between heterosexual and LGB+ respondents, ages 51-64

**eTable 10.** Mean disparities in the top ten "high disparity" states between heterosexual and LGB+ respondents, ages 51-55

**eTable 11.** Medicare eligibility age-related discontinuities in coverage, access, and self-reported health by sexual orientation in "high disparity" states, based on respondents aged 51-55

**eTable 12.** Medicare eligibility age-related discontinuities in coverage, access, and self-reported health among married individuals by sexual orientation at the national level

**eTable 13.** Medicare eligibility age-related discontinuities in coverage, access, and self-reported health by sexual orientation in the period post-Obergefell v. Hodges at the national level

**eTable 14.** Medicare eligibility age-related discontinuities in coverage, access, and self-reported health by gender identity

**eTable 15.** Medicare eligibility age-related discontinuities in coverage, access, and self-reported health by sexual orientation, weighted

**eTable 16.** Medicare eligibility age-related discontinuities in coverage, access, and self-reported health by gender identity, weighted

**eTable 17.** Robustness of Medicare eligibility age-related discontinuities to alterations in the statistical model

This supplemental material has been provided by the authors to give readers additional information about their work.

© 2024 Gavulic KA et al. *JAMA Health Forum.*

## eMethods. Supplemental Description of Methods

A. Additional background on Medicare eligibility as an age-based discontinuity

Our study leverages the natural experiment at age 65 that arises because Medicare is a (nearly) universal health insurance program that primarily provides health insurance for people age 65 or older, but also provides coverage for some younger adults with disabilities, end stage renal disease, and amyotrophic lateral sclerosis (ALS). For those who were not eligible for Medicare prior to age 65, the near-universality of Medicare eligibility at age 65 creates a sharp age-based discontinuity leading to: (a) a large shift in the primary source of health insurance coverage from other sources (e.g., commercial, Medicaid) to Medicare; and (b) an increase in the overall share of the population that has some form of health insurance. Numerous studies have leveraged this discontinuity to estimate the causal effects of gaining eligibility for Medicare coverage.

B. Regression discontinuity visualizations

For transparency, we present scatterplots of the age trend in our primary and secondary outcomes by sexual orientation and gender identity. For illustrative purposes, in each chart we plot a line of best fit based on a local regression model that uses the optimal bandwidth selected by the RD Honest model for each outcome separately by group. For our primary and secondary outcomes by sexual orientation and descriptive characteristics plots, these lines of best fit were based on the default kernel (epanechikov) and polynomial smoothing degree 2. For plots where the underlying sample sizes were smaller (i.e., the figures split by gender identity, region or those limited to the top 10 states) the lines of best fit were based on the default kernel and a polynomial smoothing degree 1 to avoid the appearance of overfitting. As a reminder, these lines of best fit were for illustrative purposes only and our primary regression discontinuity results are based on the RD Honest local linear regression model with a uniform kernel.

C. Exclusions from the study sample and missing data

In addition to excluding those who responded, “don’t know,” “not sure,” or “refused” to both the sexual orientation and gender identity questions, we excluded individuals who were not asked or missing data for the sexual orientation and gender identity. As a reminder, respondents who provided sexual orientation but not gender identity, or vice versa, were included. We did not remove respondents who had missing outcomes data but rather for any

© 2024 Gavulic KA et al. *JAMA Health Forum.*

outcome that a respondent's value is "missing," we allowed that person to naturally drop out of that model and demonstrate that the missingness on our primary outcomes is not discontinuous at age 65 (eTable 1).

Our primary sample was limited to respondents ages 51-79 years in 43 US states. We excluded the following states that did not report SOGI data in the BRFSS for any of our study years: Alabama, Maine, Nebraska, New Hampshire, North Dakota, Oregon, and South Dakota. Due to its lack of SOGI data collection during our study period, we also did not include the District of Columbia.

D. Statistical model for estimating differences-in-disparities at age 65

In the manuscript, we use RD Honest to estimate the adjusted discontinuity in each of our outcome variables at the Medicare Eligibility Age threshold. RD Honest uses local linear regression, but the linear regression analog is:

$$Y_{it} = \beta_0 + \beta_1 \times Age_{it} + \beta_2 \times Post_{65} + \beta_3 \times Age_{it} \times Post_{65} + \epsilon_{it}$$

where $Y_{it}$ is the outcome for individual *i* when they are *t* years-old, $Post_{65}$ is a dummy variable indicating whether an individual had attained the Medicare Eligibility Age of 65, $Age_{it}$ is an individual's age in years, and $\epsilon_{it}$ is noise. Including $Age_{it}$ and $Age_{it} \times Post_{65}$ allows linear age trends in the outcome to differ for individuals above and below the Medicare Eligibility Age. For a tractable approach to estimating the change in LGB+ disparities at age 65, we use linear regression (rather than RD Honest) and modify the model as follows:

$$Y_{it} = LGB_i \times (\alpha + Age_{it} + Post_{65} + Age_{it} \times Post_{65} + X_i + \gamma_t) + \epsilon_{it}$$

Where $LGB_i$ is an indicator that a respondent identified as a sexual minority, including lesbian, gay, bisexual, or "other." The coefficient on the term $LGB_i$ would capture the difference in the mean outcome between LGB+ and heterosexual respondents. The coefficient of interest is on the term $LGB_i \times Post_{65}$, which measures the adjusted discontinuity in the disparity between LGB+ and heterosexual respondents at age 65. We once again include $Age_{it}$ and $Age_{it} \times Post_{65}$ terms to allow linear age trends in the outcome to differ for individuals above and below the Medicare Eligibility Age. Due to small sample sizes of transgender and gender diverse people, we did not repeat this approach to compute differences-in-discontinuities by gender identity.

E. Additional description of secondary analyses among "high disparity" states

In secondary analyses, among the "high disparity" states, we explored mean state-level disparities for respondents ages 51-64 by sexual orientation for our four primary outcomes: 1) share insured; 2) share with usual source of care;

© 2024 Gavulic KA et al. *JAMA Health Forum.*

3) share reporting cost barriers to care; and 4) share in good or better health. We used the state-level disparities to create state-specific composite scores (based on mean difference between heterosexual and LGB+ adults in these four outcomes), allowing us to rank states and identify the top ten "high disparity" states (eTable 9). Then, we reran our RDD for all study outcomes on this subset of "high disparity" states. To test the robustness of our results among "high disparity" states, we repeated this procedure on an alternative definition of "high disparity" states, i.e. based on state-level differences among heterosexual and LGB+ people ages 51-55 years (eTable 10). State-level analyses were not completed for gender minority individuals given small sample sizes.

© 2024 Gavulic KA et al. *JAMA Health Forum.*

**eFigure 1. Medicare eligibility age-related discontinuities in access and self-reported health by sexual orientation**

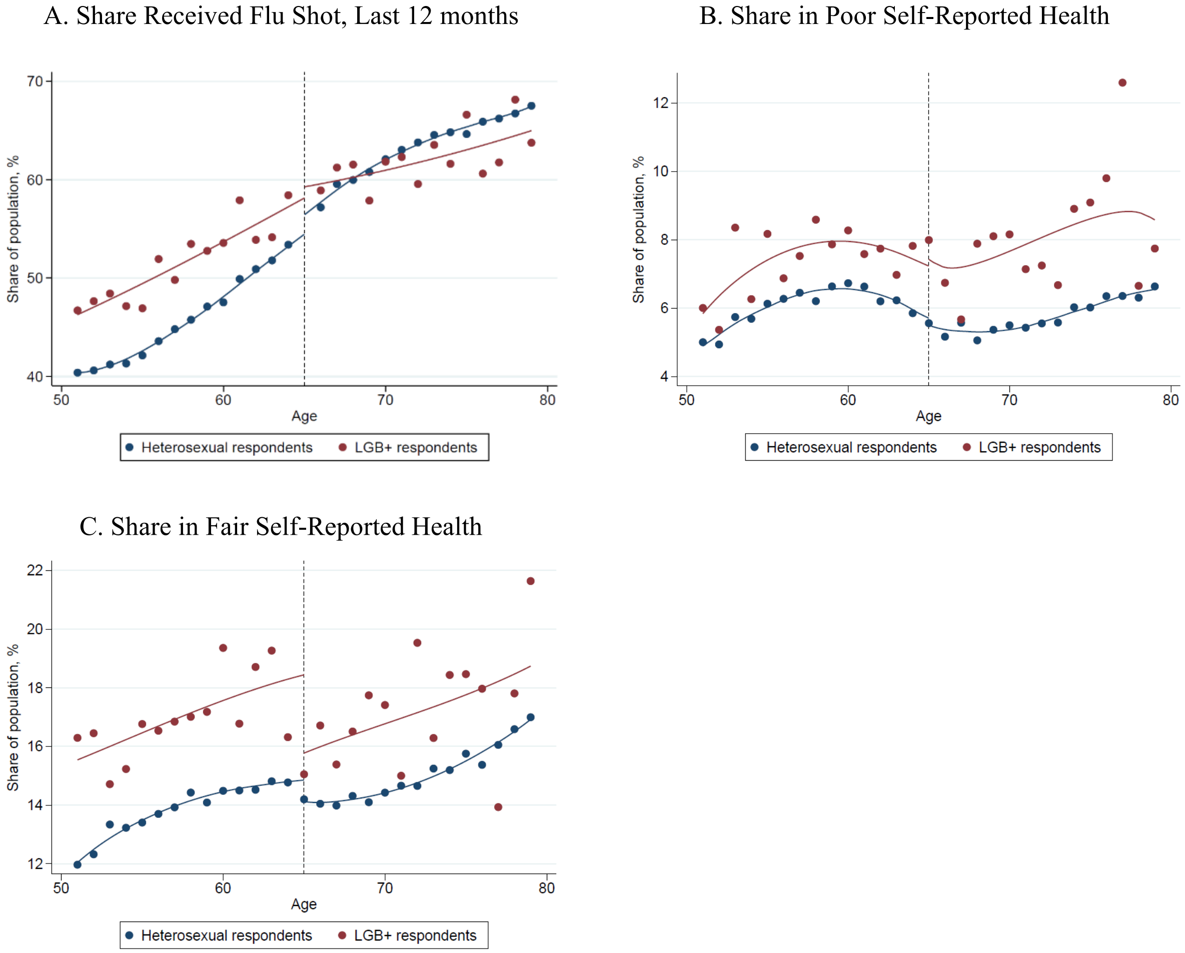


Note: For each panel, we plot the share of the study population reporting each secondary outcome by age in years separately for heterosexual and LGB+ respondents during the study period (2014-2021). For illustrative purposes, the line of best fit is based on a local regression model using the optimal bandwidth selected by the RD Honest model for each outcome separately for heterosexual and LGB+ respondents (see **eMethods** in **Supplement**). The Medicare eligibility age threshold at 65 years is represented by the dotted black line.

© 2024 Gavulic KA et al. *JAMA Health Forum.*

## eFigure 2. Medicare eligibility age-related discontinuities in access and self-reported health by sexual orientation in the top ten “high disparity” states

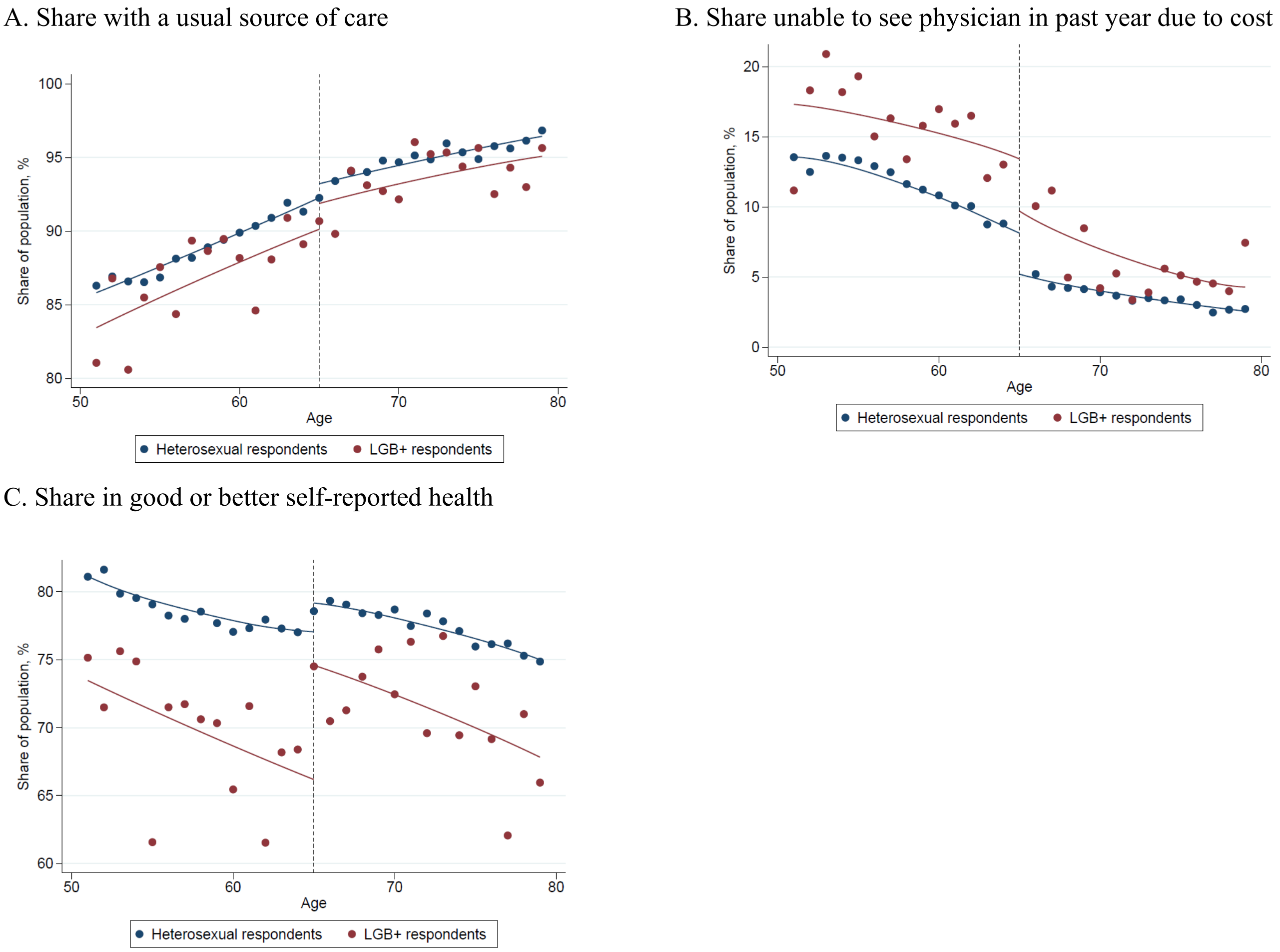


Note: For each panel, we plot the share of the study population, in the top ten “high disparity” states, that report each outcome by age in years separately for heterosexual and LGB+ respondents during the study period (2014-2021). The top ten “high disparity” states based on the mean disparity among the near-elderly (51-64 year-olds) were Mississippi, Tennessee, Kansas, Texas, Missouri, Oklahoma, Pennsylvania, New Jersey, Iowa, and New Mexico. For illustrative purposes, the line of best fit is based on a local regression model using the optimal bandwidth selected by the RD Honest model for each outcome separately for heterosexual and LGB+ respondents (see **eMethods** in **Supplement**). The Medicare eligibility age threshold at 65 years is represented by the dotted black line.

© 2024 Gavulic KA et al. *JAMA Health Forum.*

**eFigure 3. Medicare eligibility age-related discontinuities in coverage, access and self-reported health by sexual orientation in the United States Northeast**

A. Shared Insured

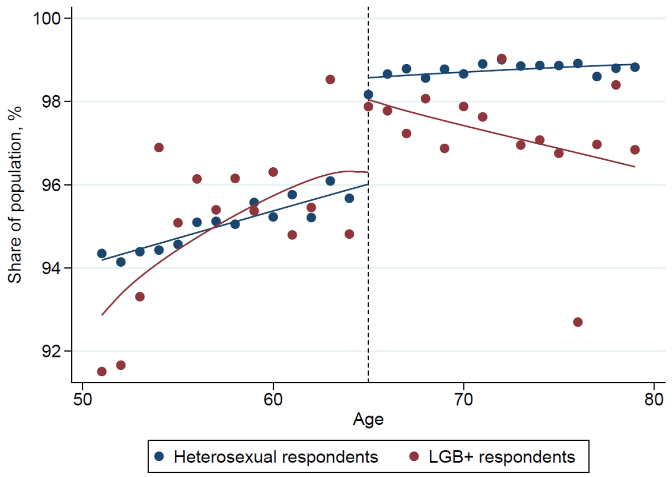


B. Share with a usual source of care

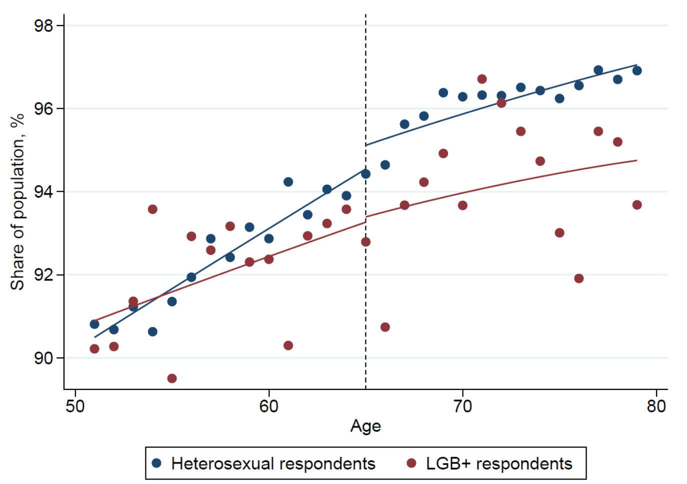


C. Share unable to see physician in past year due to cost

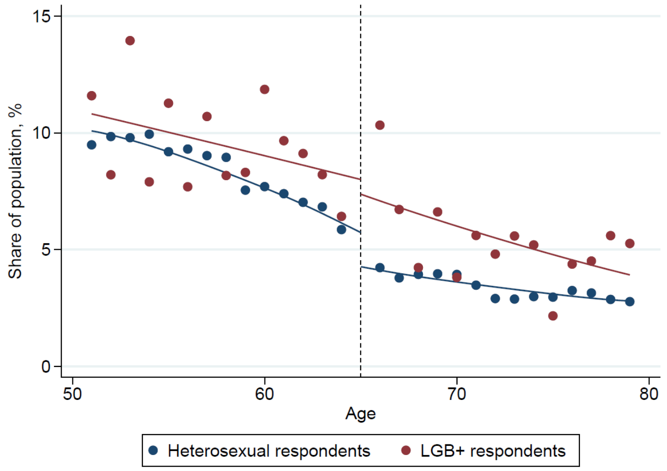


D. Share in good or better self-reported health

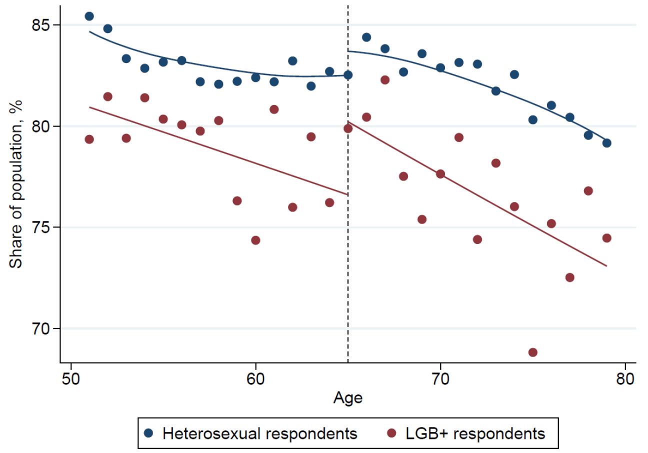


Note: For each panel, we plot the share of the study population, in the Northeastern census region of the United States, that report each outcome by age in years separately for heterosexual and LGB+ respondents during the study period (2014-2021). The states comprising the US Northeast include: Connecticut, Massachusetts, Rhode Island, Vermont, New York, New Jersey, and Pennsylvania (Maine and New Hampshire were excluded from our sample due to not reporting SOGI data in the BRFSS for any of our study years). For illustrative purposes, the line of best fit is based on a local regression model using the optimal bandwidth selected by the RD Honest model for each outcome separately for heterosexual and LGB+ respondents (see **eMethods** in Supplement). The Medicare eligibility age threshold at 65 years is represented by the dotted black line.

© 2024 Gavulic KA et al. *JAMA Health Forum.*

**eFigure 4. Medicare eligibility age-related discontinuities in coverage, access and self-reported health by sexual orientation in the United States Midwest**

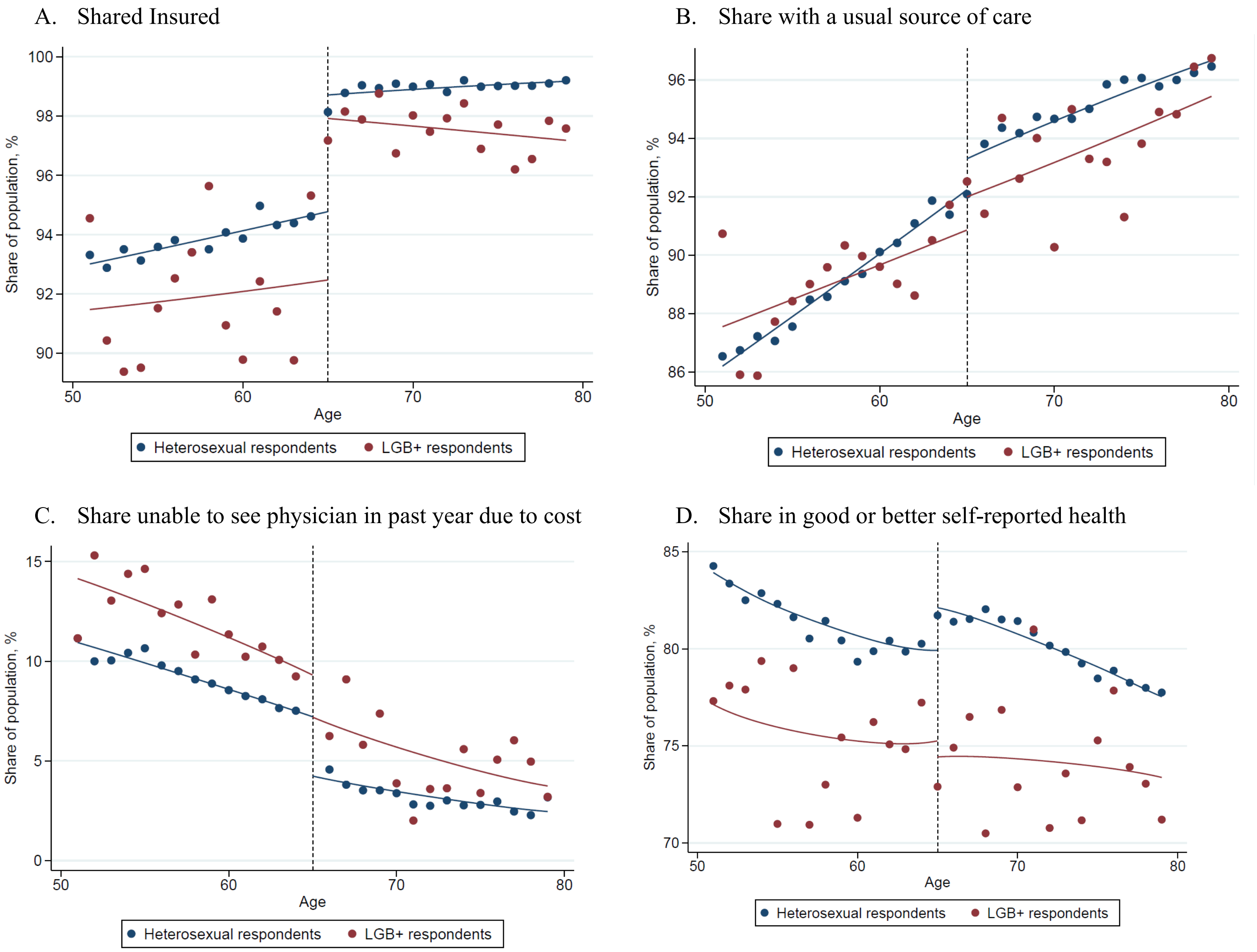


Note: For each panel, we plot the share of the study population, in the Midwestern census region of the United States, that report each outcome by age in years separately for heterosexual and LGB+ respondents during the study period (2014-2021). The states comprising the US Midwest include: Indiana, Illinois, Michigan, Ohio, Wisconsin, Iowa, Kansas, Minnesota, and Missouri (Nebraska, North Dakota, and South Dakota were excluded from our sample due to not reporting SOGI data in the BRFSS for any of our study years). For illustrative purposes, the line of best fit is based on a local regression model using the optimal bandwidth selected by the RD Honest model for each outcome separately for heterosexual and LGB+ respondents (see **eMethods** in Supplement). The Medicare eligibility age threshold at 65 years is represented by the dotted black line.

© 2024 Gavulic KA et al. *JAMA Health Forum.*

**eFigure 5. Medicare eligibility age-related discontinuities in coverage, access and self-reported health by sexual orientation in the United States South**

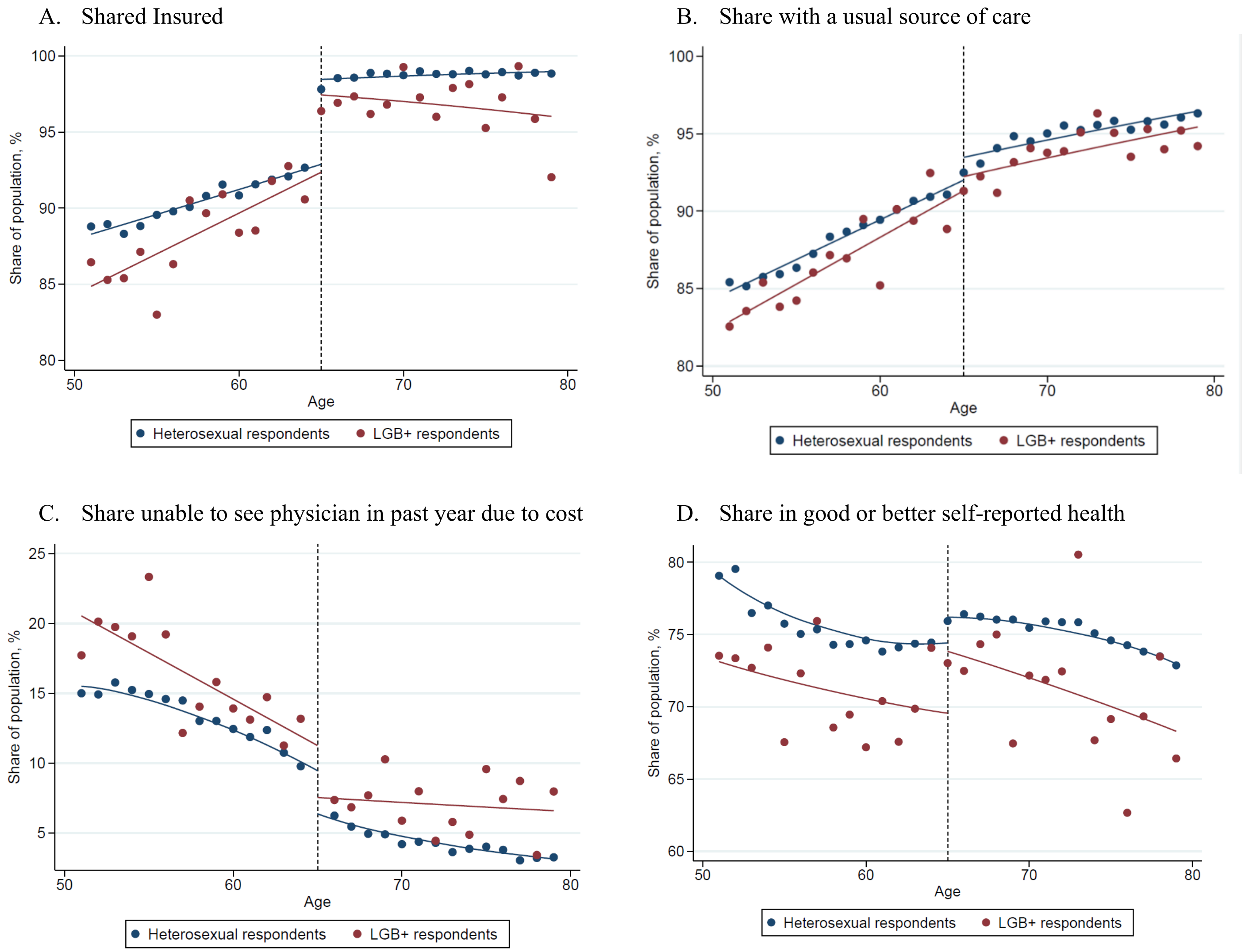


Note: For each panel, we plot the share of the study population, in the Southern census region of the United States, that report each outcome by age in years separately for heterosexual and LGB+ respondents during the study period (2014-2021). The states comprising the US South include: Delaware, Maryland, West Viriginia, Virginia, North Carolina, South Carolina, Georgia, Florida, Mississippi, Tennessee, Kentucky, Texas, Oklahoma, Arkansas, and Louisiana (Alabama and the District of Columbia were excluded from our sample due to not reporting SOGI data in the BRFSS for any of our study years). For illustrative purposes, the line of best fit is based on a local regression model using the optimal bandwidth selected by the RD Honest model for each outcome separately for heterosexual and LGB+ respondents (see **eMethods** in Supplement). The Medicare eligibility age threshold at 65 years is represented by the dotted black line.

© 2024 Gavulic KA et al. *JAMA Health Forum.*

**eFigure 6. Medicare eligibility age-related discontinuities in coverage, access and self-reported health by sexual orientation in the United States West**

A. Shared Insured

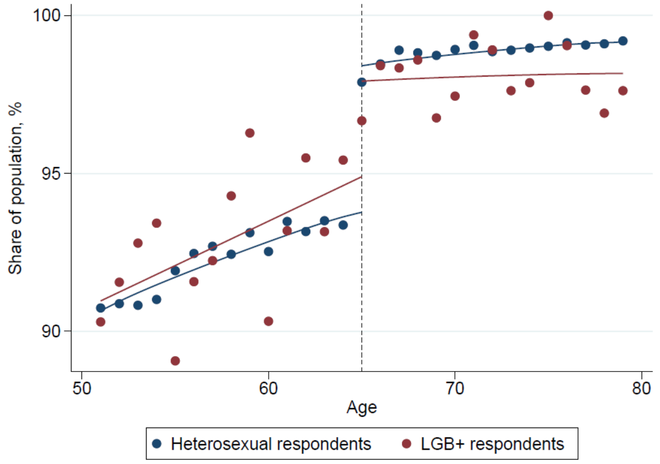


B. Share with a usual source of care

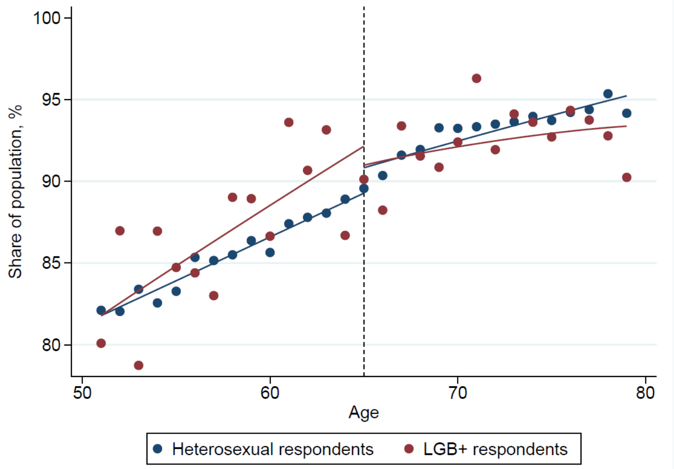


C. Share unable to see physician in past year due to cost

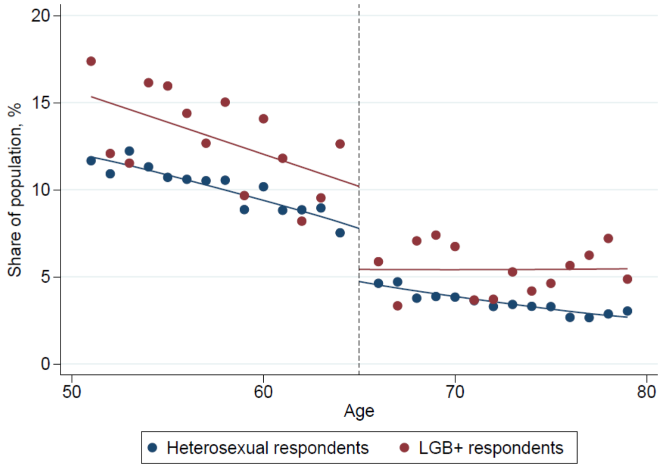


D. Share in good or better self-reported health

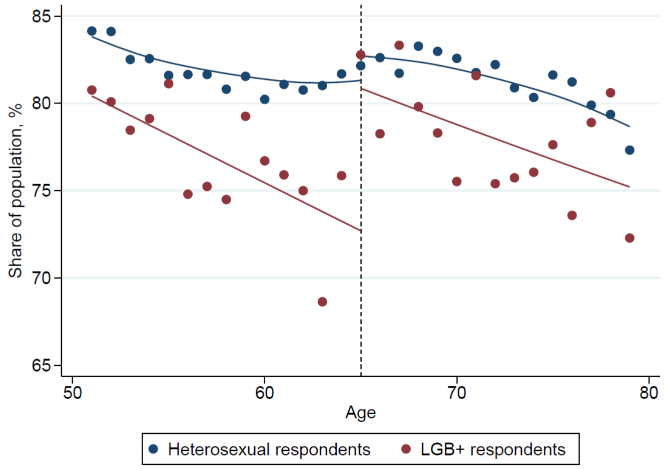


Note: For each panel, we plot the share of the study population, in the Western census region of the United States, that report each outcome by age in years separately for heterosexual and LGB+ respondents during the study period (2014-2021). The states comprising the US West include: Montana, Idaho, Wyoming, Nevada, Utah, Colorado, Arizona, New Mexico, California, Washington, Alaska, and Hawaii (Oregon was excluded from our sample due to not reporting SOGI data in the BRFSS for any of our study years). For illustrative purposes, the line of best fit is based on a local regression model using the optimal bandwidth selected by the RD Honest model for each outcome separately for heterosexual and LGB+ respondents (see **eMethods** in Supplement). The Medicare eligibility age threshold at 65 years is represented by the dotted black line.

© 2024 Gavulic KA et al. *JAMA Health Forum.*

## eFigure 7. Medicare eligibility age-related discontinuities in coverage, access, and self-reported health by gender identity

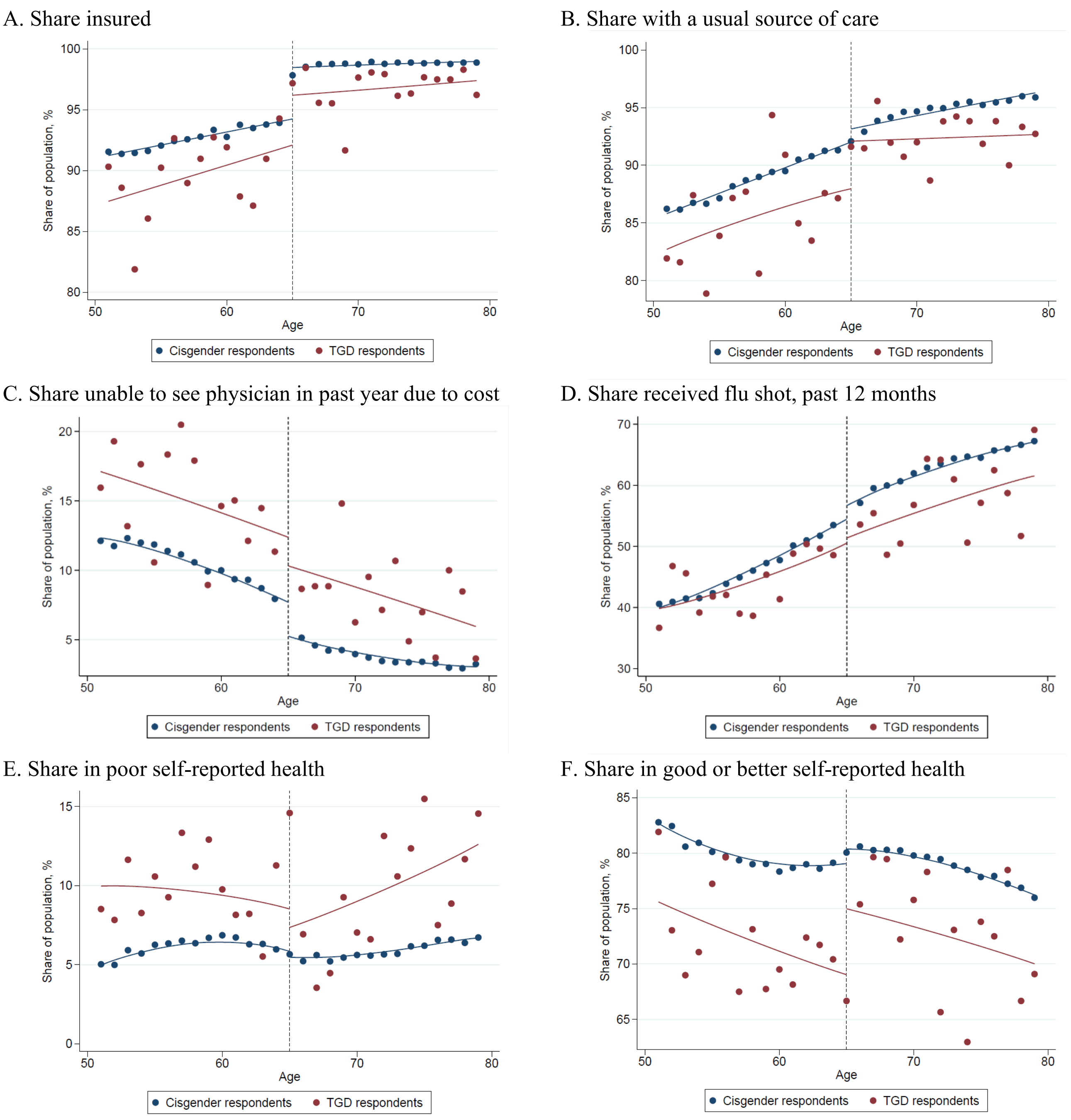


Note: For each panel, we plot the share of the study population reporting each outcome by age in years separately for cisgender and TGD respondents during the study period (2014-2021). For illustrative purposes, the line of best fit is based on a local regression model using the optimal bandwidth selected by the RD Honest model for each outcome separately for cisgender and TGD respondents (see **eMethods** in Supplement). The Medicare eligibility age threshold at 65 years is represented by the dotted black line.

© 2024 Gavulic KA et al. *JAMA Health Forum.*

## eFigure 8. Covariate smoothness test for select study population characteristics

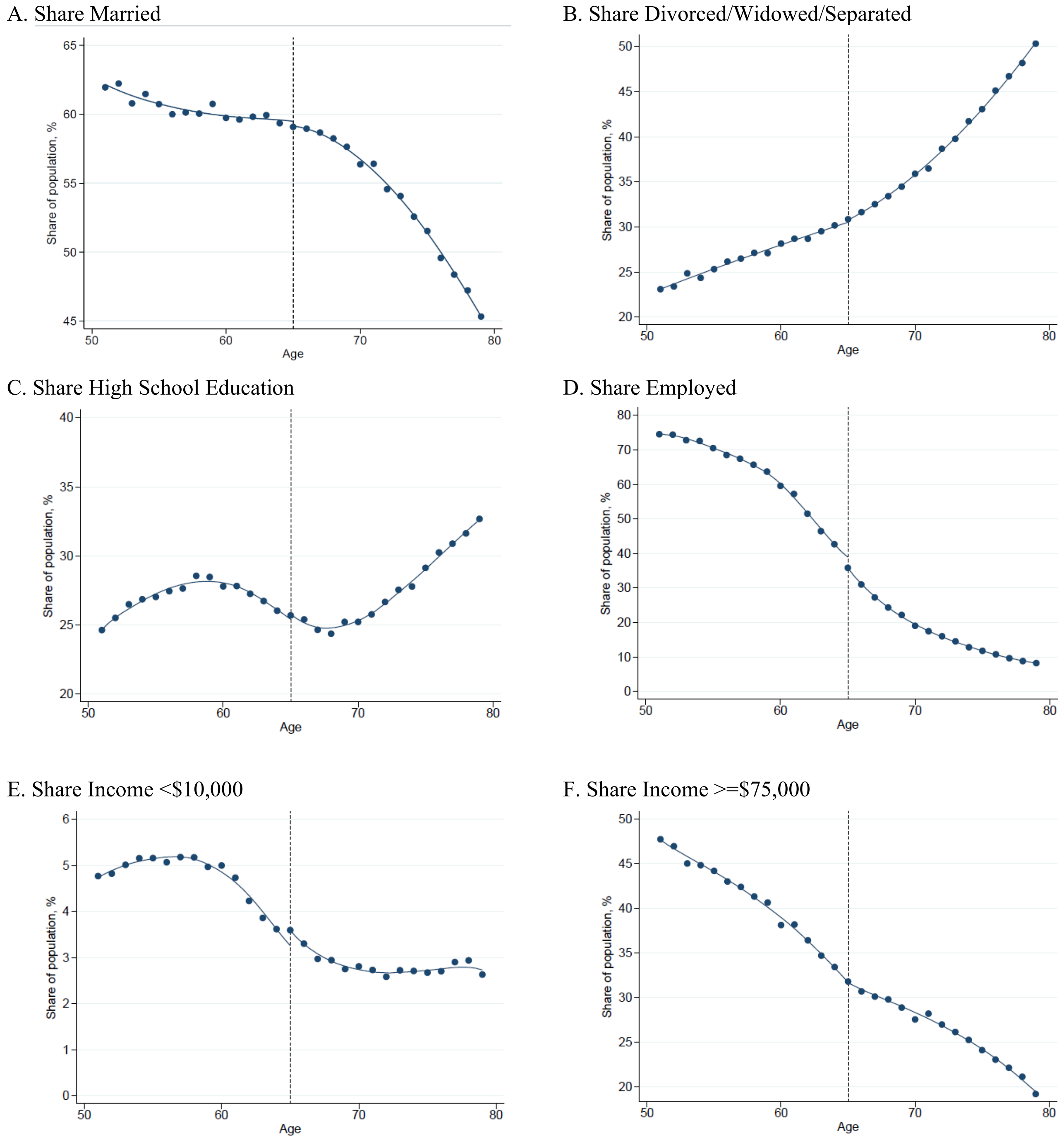


Note: For each panel, we plot the share of the study population reporting each characteristic by age in years during the study period (2014-2021). For illustrative purposes, the line of best fit is based on a local regression model using the optimal bandwidth selected by the RD Honest model for each outcome (see **eMethods** in **Supplement**). The Medicare eligibility age threshold at 65 years is represented by the dotted black line.

© 2024 Gavulic KA et al. *JAMA Health Forum.*

## eFigure 9. Smoothness tests for compositional change in reported sexual orientation and gender identity

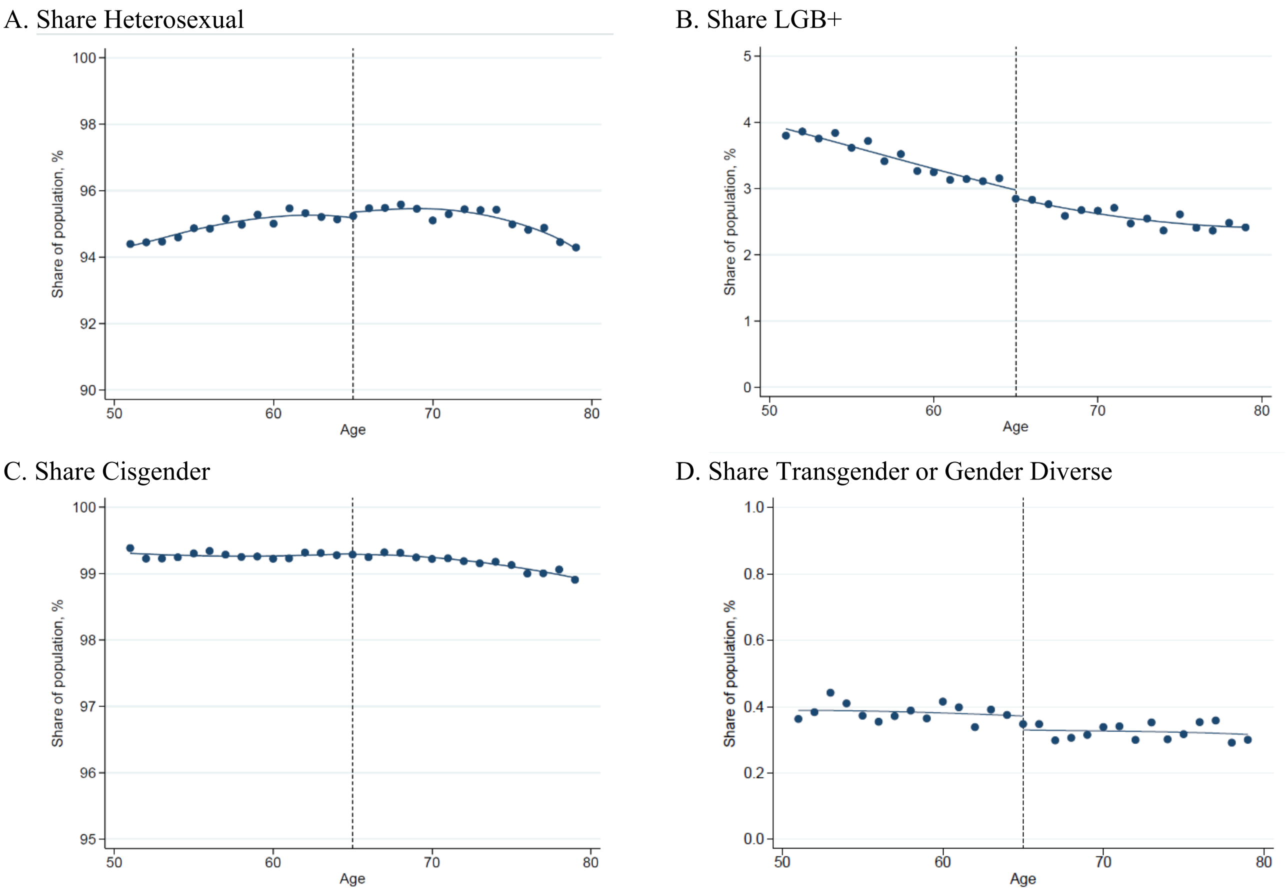


Note: For each panel, we plot the share of the study population reporting each characteristic by age in years during the study period (2014-2021). For illustrative purposes, the line of best fit is based on a local regression model using the optimal bandwidth selected by the RD Honest model for each outcome (see **eMethods** in **Supplement**). The Medicare eligibility age threshold at 65 years is represented by the dotted black line.

© 2024 Gavulic KA et al. *JAMA Health Forum.*

**eFigure 10. Smoothness tests for the number of respondents by sexual orientation**

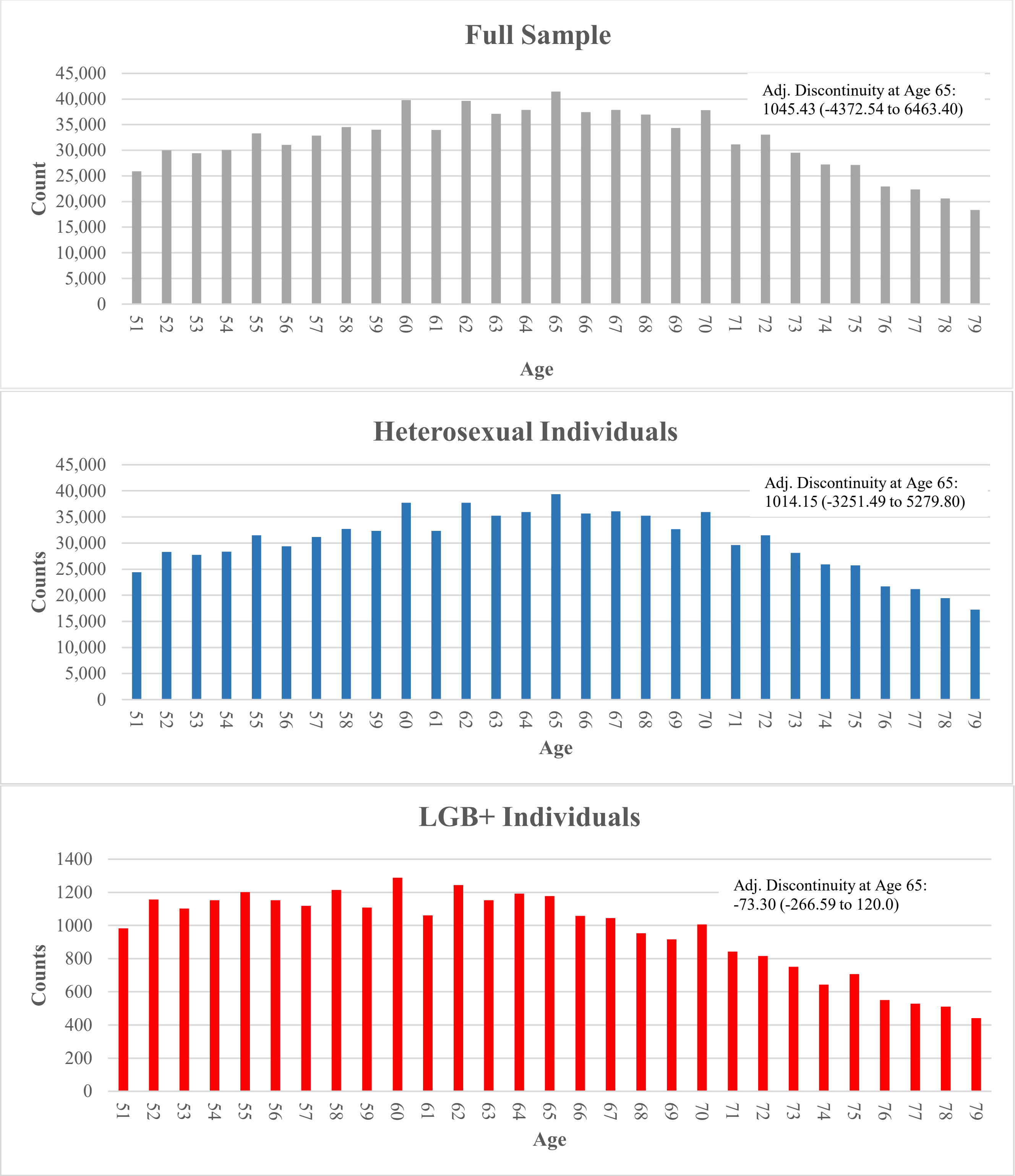


Note: The number of respondents in the overall sample and in each subgroup by sexual orientation are collapsed by age across years 2014-2021 and plotted for ages 51-79. We use the RDHonest package, consistent with our regression discontinuity approach for the primary and secondary analyses, to assess whether there were any discontinuities in the number of numbers at age 65 in our overall sample or for any subgroup. The reported point estimate reflects the discontinuity at age 65 and 95% confidence intervals are bias-adjusted from RDHonest.

© 2024 Gavulic KA et al. *JAMA Health Forum.*

**eFigure 11. Smoothness tests for the number of respondents by gender identity**

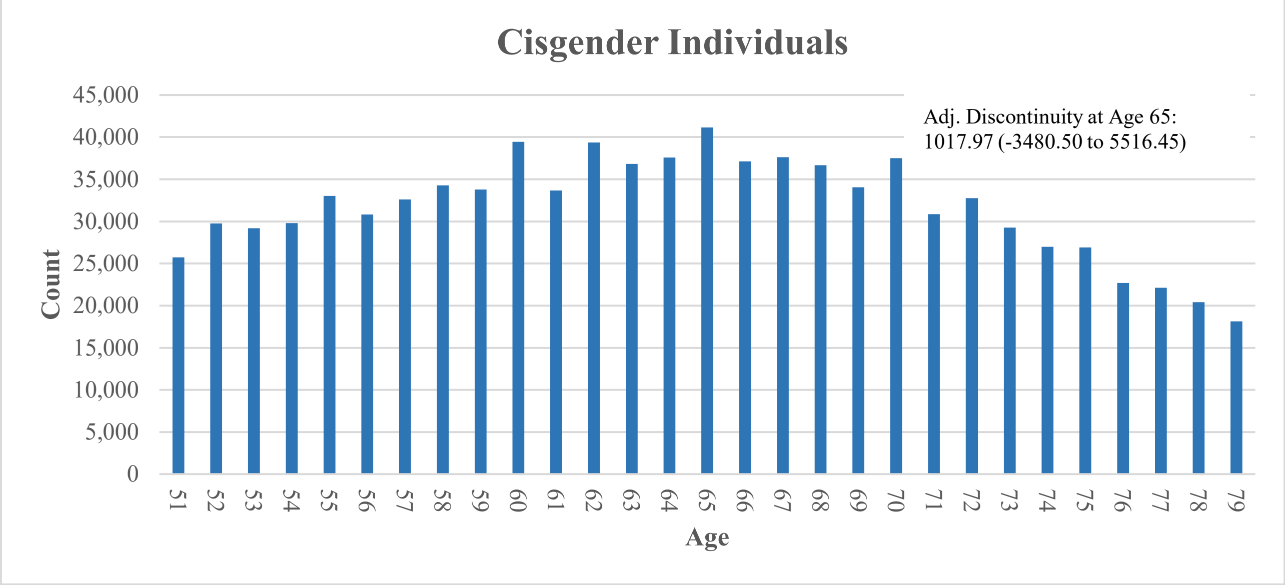


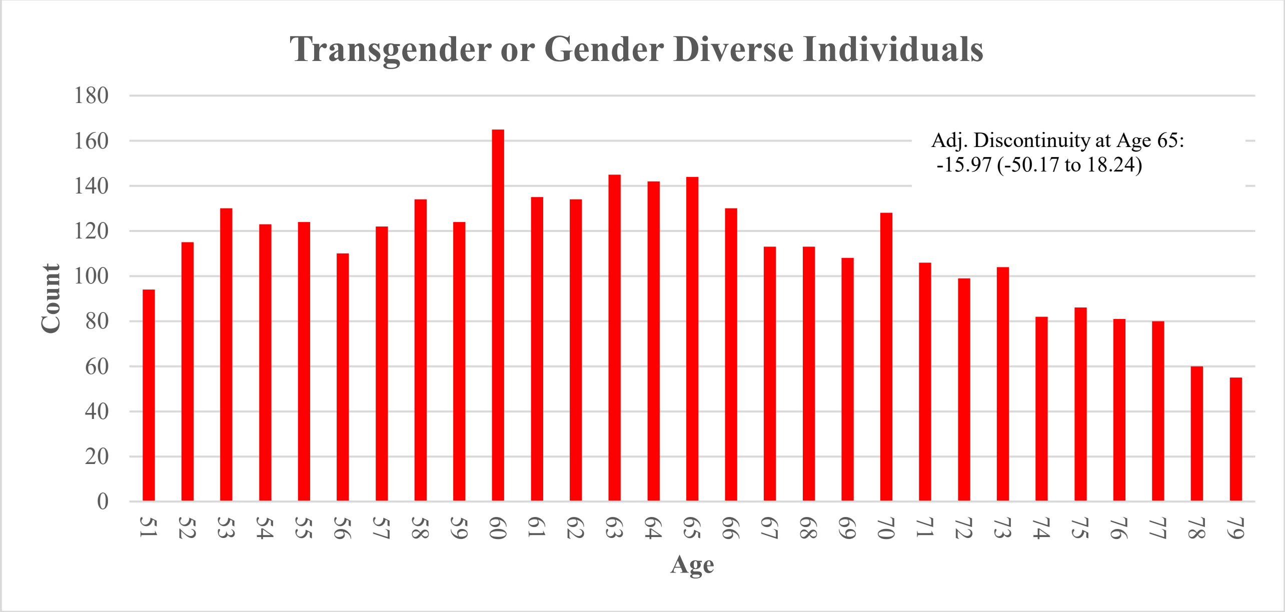


Note: The number of respondents by gender identity are collapsed by age across years 2014-2021 and plotted for ages 51-79. We use the RDHonest package, consistent with our regression discontinuity approach for the primary and secondary analyses, to assess whether there were any discontinuities in the number of numbers at age 65 in our overall sample or for any subgroup. The reported point estimate reflects the discontinuity at age 65 and 95% confidence intervals are bias-adjusted from RDHonest.

© 2024 Gavulic KA et al. *JAMA Health Forum.*

## eTable 1. Missingness in primary outcomes

| | Change at age 65 years | |
|---|---|---|
| | **Expected Mean, %[a]** | **Adjusted discontinuity (95% CI)[b]** |
| ***Full Sample*** | | |
| In insurance coverage, % | 99.3 | 0.16 (0.03 to 0.29) |
| In usual source of care, % | 99.6 | 0.02 (-0.05 to 0.10) |
| In cost barriers to seeing a physician in the past year, % | 99.8 | 0.01 (-0.07 to 0.09) |
| In good or better self-reported health, % | 99.7 | 0.01 (-0.05 to 0.06) |

[a] Column contains the expected mean at age 65 years, which is based on the local linear association between age and the outcome in each row. The expected means contain the counterfactual outcome at age 65 years in the absence of treatment (the expected outcome at 65 years without Medicare).

[b] Adjusted discontinuity estimates are in percentage points and report the RD Honest estimates and bias-adjusted confidence intervals. We use the same approach to estimate the discontinuities in the missingness of our primary outcomes as we use to estimate the discontinuities of our outcomes in our main analyses.

© 2024 Gavulic KA et al. *JAMA Health Forum.*

**eTable 2. Weighted study pop. characteristics and Medicare age-related discontinuities**

| | Sample means, %[a] | | Change at age 65 years | |
|---|---|---|---|---|
| Characteristic | Ages 51-64 | Ages 65-79 | Expected mean, %[b] | Adjusted discontinuity (95% CI)[c] |
| **Sex** | | | | |
| Male | 48.1 | 45.3 | 47.4 | 1.02 (-0.23 to 2.27) |
| Female | 51.9 | 54.7 | 52.6 | -0.99 (-2.23 to 0.26) |
| Don't know/not sure/refused | 0.0 | 0.0 | 0.1 | -0.02 (-0.07 to 0.02) |
| **Gender** | | | | |
| Cisgender | 99.1 | 99.0 | 99.3 | -0.14 (-0.43 to 0.15) |
| Transgender or gender diverse | 0.4 | 0.4 | 0.3 | 0.07 (-0.13 to 0.26) |
| Don't know/not sure/refused | 0.5 | 0.7 | 0.4 | 0.10 (-0.12 to 0.32) |
| **Sexual Orientation** | | | | |
| Heterosexual | 94.3 | 94.5 | 94.7 | 0.37 (-0.10 to 0.83) |
| LGB+ | 3.4 | 2.6 | 3.0 | -0.28 (-0.60 to 0.04) |
| Don't know/not sure/refused | 2.3 | 2.9 | 2.4 | 0.02 (-0.40 to 0.44) |
| **Marital Status** | | | | |
| Married | 63.4 | 59.8 | 62.9 | 1.31 (0.30 to 2.32) |
| Member of unmarried couple | 2.4 | 1.2 | 1.7 | -0.07 (-0.41 to 0.27) |
| Divorced/widowed/separated | 24.4 | 33.7 | 27.9 | -1.42 (-2.19 to -0.66) |
| Never married | 9.8 | 5.3 | 7.5 | 0.07 (-0.61 to 0.75) |
| **Employed** | 62.5 | 19.1 | 37.7 | -2.83 (-5.10 to -0.56) |
| **Education** | | | | |
| Less than high school | 12.4 | 12.9 | 11.5 | -0.12 (-1.50 to 1.26) |
| High school graduate | 28.1 | 28.4 | 27.6 | -0.08 (-1.59 to 1.44) |
| Some college | 30.7 | 31.1 | 32.7 | -0.17 (-1.45 to 1.12) |
| College graduate | 28.8 | 27.6 | 28.2 | 0.56 (-0.89 to 2.01) |
| **Income, $** | | | | |
| <10, 000 | 5.0 | 3.6 | 3.6 | 0.64 (-0.25 to 1.52) |
| 10 000 - 24 999 | 17.8 | 22.6 | 21.1 | -1.55 (-2.38 to -0.72) |
| 25 000 - 49 999 | 20.3 | 29.5 | 24.9 | 1.50 (-0.16 to 3.16) |
| 50 000 - 74 999 | 15.7 | 17.2 | 17.3 | 0.50 (-0.45 to 1.46) |
| >= 75 000 | 41.3 | 27.1 | 32.3 | -0.95 (-2.45 to 0.55) |
| **Race** | | | | |
| NH Black | 12.2 | 10.0 | 10.9 | 0.40 (-0.55 to 1.34) |
| Hispanic | 11.4 | 7.5 | 8.5 | 0.54 (-0.55 to 1.64) |
| NH White | 70.1 | 77.4 | 74.7 | -1.36 (-3.25 to 0.53) |
| NH Other races | 6.3 | 5.1 | 4.7 | 1.21 (0.05 to 2.38) |

[a] Columns contain weighted proportions in percentages (raw counts are available in **Table 1**).
[b] Column contains the expected mean at age 65 years, which is based on the local linear association between age and the outcome in each row. The expected means contain the counterfactual outcome at age 65 years in the absence of treatment (the expected outcome at 65 years without Medicare).
[c] Adjusted discontinuity estimates are in percentage points and report the RD Honest estimates and bias-adjusted confidence intervals (refer to **Methods** sections for more detail on the statistical model).

© 2024 Gavulic KA et al. *JAMA Health Forum.*

**eTable 3. Demographic characteristics of the study population by sexual orientation**

| | Sample means, No. (%)[a] | | | | | |
|---|---|---|---|---|---|---|
| | Age, 51-64 y | | P value[d] | Age, 65-79 y | | P value[d] |
| Characteristic | HE[b] | LGB+[c] | | HE[b] | LGB+[c] | |
| **Sex** | | | | | | |
| Male | 197,958 (44.50) | 7,992 (49.55) | <0.001 | 183,402 (42.12) | 6,023 (50.41) | <0.001 |
| Female | 246,795 (55.48) | 8,121 (50.35) | | 251,909 (57.85) | 5,912 (49.48) | |
| Don't know/not sure/refused | 118 (0.03) | 16 (0.10) | | 108 (0.02) | 13 (0.11) | |
| **Gender** | | | | | | |
| Cisgender | 441,793 (99.37) | 15,598 (96.79) | <0.001 | 431,852 (99.27) | 11,482 (96.21) | <0.001 |
| Transgender or gender diverse | 1,354 (0.30) | 377 (2.34) | | 1,147 (0.26) | 275 (2.30) | |
| Don't know/not sure/refused | 1,452 (0.33) | 141 (0.87) | | 2,032 (0.47) | 177 (1.48) | |
| **Marital Status** | | | | | | |
| Married | 271,562 (61.40) | 5,817 (36.45) | <0.001 | 241,011 (55.60) | 4,371 (36.93) | <0.001 |
| Member of unmarried couple | 8,653 (1.96) | 1,363 (8.54) | | 4,494 (1.04) | 626 (5.29) | |
| Divorced/widowed/separated | 119,072 (26.92) | 3,951 (24.75) | | 164,025 (37.84) | 4,094 (34.59) | |
| Never married | 42,998 (9.72) | 4,830 (30.26) | | 23,923 (5.52) | 2,745 (23.19) | |
| **Employed** | 276,825 (62.71) | 9,412 (59.0) | <0.001 | 85,661 (19.78) | 2,443 (20.62) | 0.024 |
| **Education** | | | | | | |
| Less than high school | 26,298 (5.93) | 1,182 (7.35) | <0.001 | 25,729 (5.92) | 932 (7.82) | <0.001 |
| High school graduate | 121,077 (27.29) | 3,395 (21.10) | | 117,183 (26.97) | 2,619 (21.99) | |
| Some college | 123,766 (27.89) | 4,101 (25.49) | | 121,402 (27.95) | 2,801 (23.51) | |
| College graduate | 172,576 (38.89) | 7,412 (46.07) | | 170,102 (39.16) | 5,560 (46.68) | |
| **Income, $** | | | | | | |
| <10, 000 | 17,538 (4.56) | 974 (6.82) | <0.001 | 9,542 (2.69) | 474 (4.67) | <0.001 |
| 10 000 - 24 999 | 65,393 (17.00) | 3,231 (22.61) | | 76,258 (21.49) | 2,655 (26.13) | |
| 25 000 - 49 999 | 78,672 (20.45) | 2,938 (20.56) | | 106,791 (30.10) | 2,990 (29.43) | |
| 50 000 - 74 999 | 64,018 (16.64) | 2,119 (14.83) | | 64,300 (18.12) | 1,582 (15.57) | |
| >= 75 000 | 159,065 (41.35) | 5,028 (35.19) | | 97,890 (27.59) | 2,459 (24.20) | |
| **Race** | | | | | | |
| NH Black | 35,381 (8.08) | 1,081 (6.86) | <0.001 | 27,827 (6.49) | 713 (6.12) | <0.001 |
| Hispanic | 21,428 (4.90) | 1,116 (7.08) | | 11,631 (2.71) | 479 (4.11) | |
| NH White | 353,392 (80.75) | 12,279 (77.94) | | 367,744 (85.80) | 9,673 (83.07) | |
| NH Other races | 27,451 (6.27) | 1,279 (8.12) | | 21,426 (5.00) | 779 (6.69) | |

[a] Columns contain raw counts of respondents and unweighted proportions in percentages.
[b] HE stands for heterosexual respondents.
[c] LGB+ stands for lesbian, gay, bisexual, or another sexual minority identity respondents.
[d] P-values were obtained from Pearson chi-squared tests.

© 2024 Gavulic KA et al. *JAMA Health Forum.*

**eTable 4. Differences-in-discontinuities in study outcomes at the national level and among "high disparity" states**

| Outcome | Expected difference at 65[a] | Difference-in-discontinuity (95% CI)[b] |
|---|---|---|
| ***Panel A. Full sample*** | | |
| Insurance coverage, % | -0.23 | -0.59 (-1.58 to 0.40) |
| Health care access, % | | |
| Have a usual source of care | -0.04 | -0.98 (-2.30 to 0.34) |
| Unable to see physician in past year because of cost | 2.3 | 0.69 (-0.99 to 2.37) |
| Received an influenza vaccination in past year | 3.63 | -3.06 (-5.53 to -0.58) |
| Self-reported health, % | | |
| Poor | 1.57 | 0.20 (-1.40 to 1.80) |
| Fair | 3.94 | -1.54 (-3.26 to 0.17) |
| Good or better | -4.5 | 1.09 (-1.44 to 3.62) |
| ***Panel B. High-disparity states*** | | |
| Insurance coverage, % | -2.46 | 1.72 (-0.82 to 4.27) |
| Health care access, % | | |
| Have a usual source of care | -2.47 | 0.71 (-2.91 to 4.33) |
| Unable to see physician in past year because of cost | 5.34 | 1.14 (-3.41 to 5.68) |
| Received an influenza vaccination in past year | 4.72 | -1.62 (-11.24 to 8.01) |
| Self-reported health, % | | |
| Poor | 1.26 | 1.86 (-1.87 to 5.59) |
| Fair | 7.72 | -4.73 (-9.21 to -0.25) |
| Good or better | -9.73 | 3.11 (-2.23 to 8.46) |

[a] The expected difference subtracts the expected mean for heterosexual adults from the expected mean of LGB+ adults and represents the expected disparity by sexual orientation at age 65.
[b] The difference-in-discontinuity estimates are in percentage points and produced from a linear regression analog to RDHonest (see eMethods).

© 2024 Gavulic KA et al. *JAMA Health Forum.*

**eTable 5. Differences by sexual orientation in share insured for 51-64 year-olds by state**

| State | Sample size, N | | Share insured, % | | |
|---|---|---|---|---|---|
| | HE[a] | LGB+[b] | HE | LGB+ | Disparity[c] |
| Mississippi | 5708 | 102 | 87.99 | 79.21 | 8.78 |
| Arkansas | 2228 | 84 | 93.77 | 87.65 | 6.12 |
| West Virginia | 7553 | 185 | 93.79 | 89.07 | 4.72 |
| Texas | 16498 | 676 | 85.63 | 81.40 | 4.23 |
| Missouri | 7564 | 225 | 91.90 | 87.73 | 4.17 |
| Wisconsin | 11163 | 399 | 94.79 | 91.18 | 3.60 |
| Florida | 10817 | 388 | 84.93 | 81.35 | 3.58 |
| Kansas | 20866 | 556 | 91.79 | 88.28 | 3.51 |
| Iowa | 12369 | 297 | 95.17 | 91.86 | 3.30 |
| Indiana | 15438 | 480 | 93.39 | 90.27 | 3.11 |
| Delaware | 6164 | 323 | 93.76 | 90.71 | 3.04 |
| Illinois | 5993 | 229 | 94.46 | 91.70 | 2.75 |
| Utah | 6429 | 202 | 92.60 | 90.10 | 2.50 |
| Oklahoma | 5919 | 166 | 89.85 | 87.95 | 1.90 |
| Kentucky | 7026 | 161 | 96.18 | 94.34 | 1.84 |
| New Jersey | 4582 | 233 | 92.63 | 90.83 | 1.80 |
| Idaho | 8545 | 162 | 88.70 | 86.96 | 1.74 |
| Pennsylvania | 10520 | 308 | 94.78 | 93.11 | 1.66 |
| South Carolina | 7387 | 191 | 88.69 | 87.37 | 1.32 |
| Hawaii | 14624 | 693 | 94.49 | 93.35 | 1.14 |
| Georgia | 8626 | 308 | 87.75 | 86.84 | 0.91 |
| Minnesota | 31631 | 1094 | 94.89 | 94.02 | 0.87 |
| Alaska | 3108 | 147 | 91.29 | 90.91 | 0.38 |
| Ohio | 24949 | 749 | 93.93 | 93.67 | 0.26 |
| Virginia | 18300 | 556 | 92.82 | 92.61 | 0.20 |
| Maryland | 16535 | 516 | 95.64 | 95.53 | 0.11 |
| California | 5356 | 244 | 92.24 | 92.21 | 0.03 |
| Rhode Island | 9216 | 504 | 96.13 | 96.17 | -0.04 |
| Connecticut | 19394 | 804 | 95.90 | 95.97 | -0.07 |
| Colorado | 9682 | 410 | 92.20 | 92.36 | -0.16 |
| New York | 32270 | 1559 | 94.21 | 94.47 | -0.26 |
| Washington | 18068 | 829 | 94.59 | 94.89 | -0.30 |
| Tennessee | 2487 | 84 | 87.79 | 88.10 | -0.31 |
| Louisiana | 8955 | 279 | 90.53 | 90.88 | -0.35 |
| Massachussetts | 8905 | 487 | 96.99 | 97.53 | -0.54 |
| Arizona | 1602 | 63 | 89.79 | 90.48 | -0.68 |
| Vermont | 12796 | 670 | 95.14 | 95.93 | -0.79 |
| Montana | 9414 | 219 | 91.05 | 92.17 | -1.11 |
| Michigan | 1630 | 71 | 94.41 | 95.77 | -1.36 |
| New Mexico | 2971 | 119 | 92.12 | 94.12 | -1.99 |
| Nevada | 4592 | 197 | 90.79 | 92.86 | -2.07 |
| North Carolina | 5107 | 133 | 88.10 | 90.91 | -2.80 |
| Wyoming | 1884 | 27 | 87.96 | 92.31 | -4.35 |

[a] HE stands for heterosexual respondents.
[b] LGB+ stands for lesbian, gay, bisexual, or another sexual minority identity respondents.
[c] Disparity is the percentage point (pp) difference calculated by subtracting the mean outcome for LGB+ respondents from the mean outcome of HE respondents. States are ranked from those with the highest mean disparity to those with the lowest mean disparity. States selected as one of the top-ten "high disparity" states are highlighted in gray.

© 2024 Gavulic KA et al. *JAMA Health Forum.*

**eTable 6. Differences by sexual orientation in usual source of care for 51-64 year-olds by state**

| State | Sample size, N | | Share with a usual source of care, % | | |
|---|---|---|---|---|---|
| | HE[a] | LGB+[b] | HE | LGB+ | Disparity[c] |
| Tennessee | 2487 | 84 | 86.37 | 80.95 | 5.41 |
| New Mexico | 2971 | 119 | 83.13 | 77.97 | 5.16 |
| Arizona | 1602 | 63 | 82.24 | 77.78 | 4.46 |
| Missouri | 7564 | 225 | 89.71 | 85.27 | 4.45 |
| New Jersey | 4582 | 233 | 90.05 | 85.96 | 4.09 |
| Mississipii | 5708 | 102 | 87.91 | 84.31 | 3.60 |
| Michigan | 1630 | 71 | 93.42 | 90.14 | 3.27 |
| Maryland | 16535 | 516 | 93.27 | 90.12 | 3.15 |
| Arkansas | 2228 | 84 | 91.95 | 89.02 | 2.93 |
| Nevada | 4592 | 197 | 82.39 | 79.49 | 2.90 |
| Pennsylvania | 10520 | 308 | 93.48 | 90.85 | 2.63 |
| Florida | 10817 | 388 | 82.63 | 80.05 | 2.58 |
| Delaware | 6164 | 323 | 91.39 | 88.82 | 2.57 |
| Louisiana | 8955 | 279 | 87.77 | 85.61 | 2.16 |
| Indiana | 15438 | 480 | 91.33 | 89.47 | 1.85 |
| Connecticut | 19394 | 804 | 93.32 | 91.52 | 1.80 |
| Iowa | 12369 | 297 | 90.34 | 88.81 | 1.53 |
| Ohio | 24949 | 749 | 90.78 | 89.42 | 1.36 |
| Virginia | 18300 | 556 | 89.07 | 88.12 | 0.95 |
| Kentucky | 7026 | 161 | 90.74 | 90.00 | 0.74 |
| Georgia | 8626 | 308 | 87.71 | 87.17 | 0.54 |
| Utah | 6429 | 202 | 86.68 | 86.14 | 0.54 |
| Texas | 16498 | 676 | 84.96 | 84.44 | 0.51 |
| Rhode Island | 9216 | 504 | 94.25 | 93.84 | 0.41 |
| Washington | 18068 | 829 | 87.60 | 87.41 | 0.19 |
| Oklahoma | 5919 | 166 | 87.36 | 87.35 | 0.02 |
| North Carolina | 5107 | 133 | 87.95 | 87.97 | -0.02 |
| Hawaii | 14624 | 693 | 90.35 | 90.48 | -0.12 |
| Kansas | 20866 | 556 | 90.28 | 90.43 | -0.16 |
| New York | 32270 | 1559 | 90.95 | 91.18 | -0.23 |
| Wisconsin | 11163 | 399 | 88.75 | 89.17 | -0.42 |
| Vermont | 12796 | 670 | 92.31 | 92.79 | -0.48 |
| Illinois | 5993 | 229 | 91.01 | 91.70 | -0.69 |
| West Virginia | 7553 | 185 | 90.55 | 91.30 | -0.76 |
| South Carolina | 7387 | 191 | 87.25 | 88.95 | -1.70 |
| Alaska | 3108 | 147 | 79.04 | 80.82 | -1.78 |
| Massachussetts | 8905 | 487 | 94.82 | 96.71 | -1.88 |
| Colorado | 9682 | 410 | 85.87 | 88.21 | -2.34 |
| Minnesota | 31631 | 1094 | 85.03 | 88.17 | -3.13 |
| California | 5356 | 244 | 86.20 | 89.71 | -3.51 |
| Montana | 9414 | 219 | 80.08 | 84.02 | -3.94 |
| Idaho | 8545 | 162 | 83.44 | 88.20 | -4.76 |
| Wyoming | 1884 | 27 | 81.55 | 88.89 | -7.34 |

[a] HE stands for heterosexual respondents.
[b] LGB+ stands for lesbian, gay, bisexual, or another sexual minority identity respondents.
[c] Disparity is the percentage point (pp) difference calculated by subtracting the mean outcome for LGB+ respondents from the mean outcome of HE respondents. States are ranked from those with the highest mean disparity to those with the lowest mean disparity. States selected as one of the top-ten "high disparity" states are highlighted in gray.

© 2024 Gavulic KA et al. *JAMA Health Forum.*

**eTable 7. Differences by sexual orientation in cost barriers for 51-64 year-olds by state**

| State | Sample size, N | | Share unable to see doctor due to cost, % | | |
|---|---|---|---|---|---|
| | HE[a] | LGB+[b] | HE | LGB+ | Disparity[c] |
| Illinois | 5,993 | 229 | 9.08 | 17.03 | 7.95 |
| Michigan | 1,630 | 71 | 6.15 | 14.08 | 7.93 |
| Nevada | 4,592 | 197 | 14.71 | 22.56 | 7.85 |
| Kentucky | 7,026 | 161 | 12.58 | 19.88 | 7.29 |
| Arkansas | 2,228 | 84 | 8.19 | 14.63 | 6.44 |
| Mississipii | 5,708 | 102 | 17.36 | 23.53 | 6.17 |
| Kansas | 20,866 | 556 | 9.70 | 15.34 | 5.64 |
| Oklahoma | 5,919 | 166 | 14.50 | 20.00 | 5.50 |
| Tennessee | 2,487 | 84 | 16.48 | 21.69 | 5.21 |
| Utah | 6,429 | 202 | 9.31 | 14.36 | 5.05 |
| Hawaii | 14,624 | 693 | 7.41 | 12.41 | 5.00 |
| Texas | 16,498 | 676 | 15.89 | 20.47 | 4.59 |
| Idaho | 8,545 | 162 | 11.77 | 16.35 | 4.58 |
| New Mexico | 2,971 | 119 | 9.97 | 14.41 | 4.43 |
| Missouri | 7,564 | 225 | 11.66 | 16.07 | 4.41 |
| Colorado | 9,682 | 410 | 9.91 | 14.18 | 4.27 |
| Wyoming | 1,884 | 27 | 10.73 | 14.81 | 4.08 |
| Florida | 10,817 | 388 | 18.54 | 22.54 | 4.00 |
| North Carolina | 5,107 | 133 | 14.49 | 18.05 | 3.55 |
| New Jersey | 4,582 | 233 | 8.26 | 11.69 | 3.43 |
| Delaware | 6,164 | 323 | 10.28 | 13.71 | 3.43 |
| Virginia | 18,300 | 556 | 10.21 | 13.35 | 3.14 |
| Iowa | 12,369 | 297 | 6.53 | 9.46 | 2.93 |
| Washington | 18,068 | 829 | 9.15 | 11.86 | 2.72 |
| Minnesota | 31,631 | 1,094 | 8.17 | 10.81 | 2.64 |
| New York | 32,270 | 1,559 | 8.75 | 10.93 | 2.17 |
| Rhode Island | 9,216 | 504 | 8.17 | 9.98 | 1.81 |
| South Carolina | 7,387 | 191 | 14.67 | 16.32 | 1.64 |
| Pennsylvania | 10,520 | 308 | 9.73 | 11.36 | 1.64 |
| Ohio | 24,949 | 749 | 10.06 | 11.63 | 1.57 |
| Alaska | 3,108 | 147 | 10.16 | 11.56 | 1.40 |
| Maryland | 16,535 | 516 | 8.16 | 8.95 | 0.79 |
| Wisconsin | 11,163 | 399 | 9.51 | 10.28 | 0.76 |
| Connecticut | 19,394 | 804 | 8.01 | 8.72 | 0.71 |
| Indiana | 15,438 | 480 | 9.84 | 10.23 | 0.39 |
| Georgia | 8,626 | 308 | 15.41 | 15.64 | 0.23 |
| West Virginia | 7,553 | 185 | 11.99 | 11.96 | -0.04 |
| Vermont | 12,796 | 670 | 7.67 | 7.63 | -0.04 |
| Montana | 9,414 | 219 | 11.06 | 10.55 | -0.51 |
| Louisiana | 8,955 | 279 | 14.58 | 14.03 | -0.55 |
| Massachussetts | 8,905 | 487 | 6.90 | 5.75 | -1.16 |
| Arizona | 1,602 | 63 | 14.05 | 12.70 | -1.35 |
| California | 5,356 | 244 | 10.56 | 7.41 | -3.15 |

[a] HE stands for heterosexual respondents.
[b] LGB+ stands for lesbian, gay, bisexual, or another sexual minority identity respondents.
[c] Disparity is the percentage point (pp) difference calculated by subtracting the mean outcome for HE respondents from the mean outcome of LGB+ respondents. States are ranked from those with the highest mean disparity to those with the lowest mean disparity. States selected as one of the top-ten "high disparity" states are highlighted in gray.

© 2024 Gavulic KA et al. *JAMA Health Forum.*

**eTable 8. Differences by sexual orientation in good or better health for 51-64 year-olds by state**

| | Sample size, N | | Share in good or better health, % | | |
|---|---|---|---|---|---|
| State | HE[a] | LGB+[b] | HE | LGB+ | Disparity[c] |
| Alaska | 3,108 | 147 | 82.11 | 70.07 | 12.04 |
| Tennessee | 2,487 | 84 | 69.09 | 57.14 | 11.95 |
| Pennsylvania | 10,520 | 308 | 79.65 | 68.73 | 10.92 |
| Kansas | 20,866 | 556 | 82.21 | 72.20 | 10.01 |
| Oklahoma | 5,919 | 166 | 74.64 | 65.06 | 9.58 |
| Texas | 16,498 | 676 | 75.30 | 66.22 | 9.08 |
| Montana | 9,414 | 219 | 81.08 | 72.02 | 9.07 |
| Iowa | 12,369 | 297 | 83.16 | 75.42 | 7.74 |
| New Mexico | 2,971 | 119 | 79.84 | 72.27 | 7.58 |
| Connecticut | 19,394 | 804 | 85.78 | 78.38 | 7.40 |
| New Jersey | 4,582 | 233 | 85.87 | 78.54 | 7.33 |
| Wisconsin | 11,163 | 399 | 82.72 | 75.63 | 7.09 |
| North Carolina | 5,107 | 133 | 76.82 | 69.92 | 6.90 |
| Mississipii | 5,708 | 102 | 67.58 | 60.78 | 6.80 |
| Idaho | 8,545 | 162 | 81.18 | 74.53 | 6.64 |
| Louisiana | 8,955 | 279 | 73.29 | 66.67 | 6.62 |
| Ohio | 24,949 | 749 | 76.91 | 70.93 | 5.98 |
| Colorado | 9,682 | 410 | 84.14 | 78.24 | 5.90 |
| Washington | 18,068 | 829 | 82.08 | 76.36 | 5.72 |
| Virginia | 18,300 | 556 | 80.31 | 75.14 | 5.17 |
| South Carolina | 7,387 | 191 | 74.94 | 69.84 | 5.10 |
| Indiana | 15,438 | 480 | 77.89 | 72.80 | 5.08 |
| Hawaii | 14,624 | 693 | 82.41 | 77.34 | 5.07 |
| Illinois | 5,993 | 229 | 81.74 | 76.86 | 4.88 |
| Rhode Island | 9,216 | 504 | 82.26 | 77.49 | 4.77 |
| Missouri | 7,564 | 225 | 74.75 | 70.22 | 4.53 |
| Georgia | 8,626 | 308 | 75.77 | 71.43 | 4.34 |
| Minnesota | 31,631 | 1,094 | 85.57 | 81.54 | 4.03 |
| Kentucky | 7,026 | 161 | 69.73 | 65.84 | 3.89 |
| Utah | 6,429 | 202 | 84.23 | 81.19 | 3.04 |
| Arizona | 1,602 | 63 | 75.92 | 73.02 | 2.91 |
| Nevada | 4,592 | 197 | 77.83 | 75.13 | 2.70 |
| Massachussetts | 8,905 | 487 | 84.10 | 81.52 | 2.58 |
| Maryland | 16,535 | 516 | 82.95 | 80.39 | 2.56 |
| New York | 32,270 | 1,559 | 80.76 | 78.57 | 2.19 |
| Michigan | 1,630 | 71 | 83.60 | 81.69 | 1.91 |
| Delaware | 6,164 | 323 | 78.45 | 76.78 | 1.67 |
| Vermont | 12,796 | 670 | 85.36 | 84.16 | 1.20 |
| Florida | 10,817 | 388 | 70.14 | 70.91 | -0.76 |
| West Virginia | 7,553 | 185 | 68.58 | 69.95 | -1.37 |
| Arkansas | 2,228 | 84 | 73.08 | 78.05 | -4.97 |
| California | 5,356 | 244 | 77.63 | 83.20 | -5.57 |
| Wyoming | 1,884 | 27 | 84.57 | 92.59 | -8.03 |

[a] HE stands for heterosexual respondents.
[b] LGB+ stands for lesbian, gay, bisexual, or another sexual minority identity respondents.
[c] Disparity is the percentage point (pp) difference calculated by subtracting the mean outcome for LGB+ respondents from the mean outcome of HE respondents. States are ranked from those with the highest mean disparity to those with the lowest mean disparity. States selected as one of the top-ten “high disparity” states are highlighted in gray.

© 2024 Gavulic KA et al. *JAMA Health Forum.*

**eTable 9. Mean disparities in the top ten "high disparity" states between heterosexual and LGB+ respondents, ages 51-64**

| State | Share insured, % | | | Usual source of care, % | | | Unable to see doctor in the past year due to cost, % | | | Good or better health, % | | | Mean disparity[c] |
|---|---|---|---|---|---|---|---|---|---|---|---|---|---|
| | HE[a] | LGB+[b] | Disparity[c] | HE[a] | LGB+[b] | Disparity[c] | HE[a] | LGB+[b] | Disparity[c] | HE[a] | LGB+[b] | Disparity[c] | |
| Mississippi | 88.0 | 79.2 | 8.8 | 87.9 | 84.3 | 3.6 | 17.4 | 23.5 | 6.2 | 67.6 | 60.8 | 6.8 | 6.3 |
| Tennessee | 87.8 | 88.1 | -0.3 | 86.4 | 81.0 | 5.4 | 16.5 | 21.7 | 5.2 | 69.1 | 57.1 | 12.0 | 5.6 |
| Kansas | 91.8 | 88.3 | 3.5 | 90.3 | 90.4 | -0.2 | 9.7 | 15.3 | 5.6 | 82.2 | 72.2 | 10.0 | 4.8 |
| Texas | 85.6 | 81.4 | 4.2 | 85.0 | 84.4 | 0.5 | 15.9 | 20.5 | 4.6 | 75.3 | 66.2 | 9.1 | 4.6 |
| Missouri | 91.9 | 87.7 | 4.2 | 89.7 | 85.3 | 4.5 | 11.7 | 16.1 | 4.4 | 74.8 | 70.2 | 4.5 | 4.4 |
| Oklahoma | 89.9 | 88.0 | 1.9 | 87.4 | 87.4 | 0.0 | 14.5 | 20.0 | 5.5 | 74.6 | 65.1 | 9.6 | 4.3 |
| Pennsylvania | 94.8 | 93.1 | 1.7 | 93.5 | 90.9 | 2.6 | 9.7 | 11.4 | 1.6 | 79.7 | 68.7 | 10.9 | 4.2 |
| New Jersey | 92.6 | 90.8 | 1.8 | 90.1 | 86.0 | 4.1 | 8.3 | 11.7 | 3.4 | 85.9 | 78.5 | 7.3 | 4.2 |
| Iowa | 95.2 | 91.9 | 3.3 | 90.3 | 88.8 | 1.5 | 6.5 | 9.5 | 2.9 | 83.2 | 75.4 | 7.7 | 3.9 |
| New Mexico | 92.1 | 94.1 | -2.0 | 83.1 | 78.0 | 5.2 | 10.0 | 14.4 | 4.4 | 79.8 | 72.3 | 7.6 | 3.8 |

[a] HE stands for heterosexual respondents.
[b] LGB+ stands for lesbian, gay, bisexual, or another sexual minority identity respondents.
[c] Disparity is the percentage point (pp) difference calculated by subtracting the mean outcome for LGB+ respondents from the mean outcome of HE respondents (or the reverse in the case of the "unable to see doctor in the past year due to cost" outcome where lower values for this outcome are more favorable). The mean disparity is calculated for each state as the arithmetic mean of the pp differences for the four outcomes. States are ranked from those with the highest mean disparity to those with the lowest mean disparity.

© 2024 Gavulic KA et al. *JAMA Health Forum.*

**eTable 10. Mean disparities in the top ten "high disparity" states between heterosexual and LGB+ respondents, ages 51-55**

| | Share insured, % | | | Usual source of care,% | | | Unable to see doctor in the past year due to cost, % | | | Good or better health, % | | | |
|---|---|---|---|---|---|---|---|---|---|---|---|---|---|
| State | HE[a] | LGB+[b] | Disparity[c] | HE[a] | LGB+[b] | Disparity[c] | HE[a] | LGB+[b] | Disparity[c] | HE[a] | LGB+[b] | Disparity[c] | Mean disparity[c] |
| Wyoming | 89.2 | 80.0 | 9.2 | 78.2 | 60.0 | 18.2 | 12.3 | 40.0 | 27.7 | 88.0 | 80.0 | 8.0 | 15.8 |
| Arkansas | 91.5 | 82.6 | 8.9 | 90.4 | 79.2 | 11.2 | 10.4 | 28.0 | 17.6 | 73.6 | 83.3 | -9.7 | 7.0 |
| Oklahoma | 88.3 | 87.7 | 0.6 | 84.7 | 86.0 | -1.3 | 15.5 | 28.6 | 13.1 | 76.0 | 64.9 | 11.0 | 5.9 |
| Delaware | 92.8 | 85.9 | 7.0 | 89.2 | 82.8 | 6.4 | 11.4 | 18.4 | 7.0 | 79.3 | 76.8 | 2.5 | 5.7 |
| Texas | 82.9 | 74.6 | 8.3 | 81.8 | 81.7 | 0.0 | 18.5 | 22.9 | 4.4 | 76.5 | 67.1 | 9.4 | 5.5 |
| South Carolina | 88.2 | 80.0 | 8.2 | 84.6 | 86.3 | -1.6 | 16.4 | 25.5 | 9.0 | 76.6 | 70.6 | 6.0 | 5.4 |
| Mississippi | 85.2 | 79.1 | 6.2 | 83.6 | 84.1 | -0.5 | 20.1 | 25.0 | 4.9 | 72.0 | 61.4 | 10.6 | 5.3 |
| Pennsylvania | 93.7 | 89.8 | 3.8 | 92.0 | 87.3 | 4.7 | 11.5 | 10.1 | -1.4 | 81.0 | 69.5 | 11.5 | 4.7 |
| Alaska | 90.4 | 92.3 | -1.9 | 77.2 | 76.9 | 0.3 | 11.4 | 13.2 | 1.8 | 84.0 | 66.0 | 18.0 | 4.6 |
| Missouri | 91.3 | 92.3 | -1.1 | 87.6 | 84.0 | 3.7 | 13.1 | 17.3 | 4.1 | 77.3 | 66.7 | 10.7 | 4.4 |

[a] HE stands for heterosexual respondents.

[b] LGB+ stands for lesbian, gay, bisexual, or another sexual minority identity respondents.

[c] Disparity is the percentage point (pp) difference calculated by subtracting the mean outcome for LGB+ respondents from the mean outcome of HE respondents (or the reverse in the case of the "unable to see doctor in the past year due to cost" outcome where lower values for this outcome are more favorable). The mean disparity is calculated for each state as the arithmetic mean of the pp differences for the four outcomes. States are ranked from those with the highest mean disparity to those with the lowest mean disparity. These ten states were identified as the highest disparity states based on differences between heterosexual and LGB+ respondents aged 51-55 years old, as opposed to respondents ages 51-64 years old in eTable 9.

© 2024 Gavulic KA et al. *JAMA Health Forum.*

**eTable 11. Medicare eligibility age-related discontinuities in coverage, access, and self-reported health by sexual orientation in "high disparity" states, based on respondents aged 51-55[a]**

| | Sample Means, % | | Change at age 65 years | |
|---|---|---|---|---|
| | Ages 51-64 | Ages 65-79 | Expected mean, %[b] | Adjusted discontinuity (95% CI)[c] |
| ***Panel A. Full sample*** | | | | |
| Insurance coverage, % | 89.6 | 98.5 | 92.1 | 5.85 (5.12 to 6.58) |
| Health care access, % | | | | |
| Have a usual source of care | 87.7 | 94.3 | 91.4 | 1.08 (0.35 to 1.80) |
| Unable to see physician in past year because of cost | 13.4 | 4.4 | 8.7 | -2.38 (-3.82 to -0.93) |
| Received an influenza vaccination in past year | 45.2 | 62.7 | 54.7 | 0.40 (-1.91 to 2.71) |
| Self-reported health, % | | | | |
| Poor | 7.7 | 7.3 | 7.3 | -0.37 (-1.36 to 0.61) |
| Fair | 16.9 | 17.8 | 18.5 | -1.52 (-2.77 to -0.27) |
| Good or better | 75.4 | 75.0 | 74.3 | 1.70 (0.04 to 3.36) |
| ***Panel B. Heterosexual Individuals*** | | | | |
| Insurance coverage, % | 90.0 | 98.6 | 92.3 | 5.78 (5.05 to 6.52) |
| Health care access, % | | | | |
| Have a usual source of care | 88.0 | 94.5 | 91.5 | 1.16 (0.36 to 1.95) |
| Unable to see physician in past year because of cost | 13.1 | 4.2 | 8.5 | -2.48 (-3.94 to -1.02) |
| Received an influenza vaccination in past year | 45.2 | 62.9 | 54.7 | 0.86 (-1.48 to 3.20) |
| Self-reported health, % | | | | |
| Poor | 7.5 | 7.0 | 7.1 | -0.41 (-1.40 to 0.58) |
| Fair | 16.5 | 17.5 | 18.2 | -1.38 (-2.65 to -0.11) |
| Good or better | 76.0 | 75.6 | 74.8 | 1.63 (-0.05 to 3.30) |
| ***Panel C. Lesbian, Gay, Bisexual, or Another Sexual Minority (LGB+) Individuals*** | | | | |
| Insurance coverage, % | 86.8 | 97.0 | 91.5 | 5.94 (2.62 to 9.26) |
| Health care access, % | | | | |
| Have a usual source of care | 86.6 | 93.2 | 90.1 | -0.17 (-5.37 to 5.03) |
| Unable to see physician in past year because of cost | 16.7 | 7.5 | 11.6 | -2.14 (-8.22 to 3.93) |
| Received an influenza vaccination in past year | 51.8 | 61.1 | 61.6 | -5.35 (-15.35 to 4.65) |
| Self-reported health, % | | | | |
| Poor | 9.3 | 10.3 | 10.0 | -1.08 (-5.98 to 3.83) |
| Fair | 21.2 | 18.9 | 20.9 | -3.78 (-9.15 to 1.59) |
| Good or better | 69.5 | 70.8 | 68.8 | 5.49 (-1.99 to 12.97) |

[a] All estimates are unweighted.
[b] Column contains the expected mean at age 65 years, which is based on the local linear association between age and the outcome in each row. The expected means contain the counterfactual outcome at age 65 years in the absence of treatment (the expected outcome at 65 years without Medicare).
[c] Adjusted discontinuity estimates are in percentage points and report the RD Honest estimates and bias-adjusted confidence intervals (refer to Methods sections for more detail on the statistical model).

© 2024 Gavulic KA et al. *JAMA Health Forum.*

**eTable 12. Medicare eligibility age-related discontinuities in coverage, access, and self-reported health among married individuals by sexual orientation at the national level[a]**

| | Sample Means, % | | Change at age 65 years | |
|---|---|---|---|---|
| | Ages 51-64 | Ages 65-79 | Expected mean, % [b] | Adjusted discontinuity (95% CI) [c] |
| ***Panel A. Full married sample*** | | | | |
| Insurance coverage, % | 94.8 | 99.0 | 95.5 | 3.33 (3.02 to 3.63) |
| Health care access, % | | | | |
| Have a usual source of care | 90.9 | 95.6 | 93.5 | 0.53 (0.15 to 0.90) |
| Unable to see physician in past year because of cost | 7.8 | 2.8 | 6.1 | -2.43 (-2.86 to -2.00) |
| Received an influenza vaccination in past year | 48.3 | 65.0 | 56.3 | 2.52 (1.15 to 3.90) |
| Self-reported health, % | | | | |
| Poor | 3.8 | 4.2 | 3.9 | -0.14 (-0.54 to 0.26) |
| Fair | 10.6 | 12.5 | 12.0 | -0.77 (-1.24 to -0.30) |
| Good or better | 85.7 | 83.2 | 84.3 | 0.66 (-0.02 to 1.35) |
| ***Panel B. Heterosexual married individuals*** | | | | |
| Insurance coverage, % | 95.0 | 99.1 | 95.6 | 3.28 (2.98 to 3.58) |
| Health care access, % | | | | |
| Have a usual source of care | 91.0 | 95.7 | 93.6 | 0.63 (0.27 to 0.99) |
| Unable to see physician in past year because of cost | 7.6 | 2.7 | 6.0 | -2.42 (-2.85 to -1.99) |
| Received an influenza vaccination in past year | 48.2 | 65.2 | 56.5 | 2.43 (1.03 to 3.83) |
| Self-reported health, % | | | | |
| Poor | 3.7 | 4.2 | 3.8 | -0.12 (-0.51 to 0.28) |
| Fair | 10.4 | 12.4 | 11.8 | -0.71 (-1.19 to -0.22) |
| Good or better | 85.9 | 83.5 | 84.6 | 0.61 (-0.09 to 1.30) |
| ***Panel C. Lesbian, Gay, Bisexual, or Another Sexual Minority (LGB+) married individuals*** | | | | |
| Insurance coverage, % | 94.0 | 97.8 | 95.1 | 3.39 (1.95 to 4.83) |
| Health care access, % | | | | |
| Have a usual source of care | 91.1 | 94.3 | 94.4 | -1.91 (-4.81 to 1.00) |
| Unable to see physician in past year because of cost | 9.7 | 4.7 | 7.0 | -1.60 (-4.79 to 1.60) |
| Received an influenza vaccination in past year | 53.5 | 63.5 | 57.9 | 3.59 (-0.51 to 7.68) |
| Self-reported health, % | | | | |
| Poor | 4.3 | 5.8 | 4.8 | 0.17 (-2.19 to 2.53) |
| Fair | 12.8 | 14.8 | 15.9 | -2.81 (-6.70 to 1.08) |
| Good or better | 83.0 | 79.4 | 78.1 | 4.05 (0.48 to 7.62) |

[a] All estimates are unweighted.
[b] Column contains the expected mean at age 65 years, which is based on the local linear association between age and the outcome in each row. The expected means contain the counterfactual outcome at age 65 years in the absence of treatment (the expected outcome at 65 years without Medicare).
[c] Adjusted discontinuity estimates are in percentage points.

© 2024 Gavulic KA et al. *JAMA Health Forum.*

**eTable 13. Medicare eligibility age-related discontinuities in coverage, access, and self-reported health by sexual orientation in the period post-Obergefell v. Hodges at the national level[a]**

| | Sample Means, % | | Change at age 65 years | |
|---|---|---|---|---|
| | Ages 51-64 | Ages 65-79 | Expected mean, % [b] | Adjusted discontinuity (95% CI) [c] |
| ***Panel A. Full sample*** | | | | |
| Insurance coverage, % | 92.6 | 98.7 | 94.1 | 4.27 (4.08 to 4.46) |
| Health care access, % | | | | |
| Have a usual source of care | 88.7 | 94.5 | 91.9 | 1.00 (0.72 to 1.28) |
| Unable to see physician in past year because of cost | 10.5 | 3.9 | 7.6 | -2.12 (-2.77 to -1.47) |
| Received an influenza vaccination in past year | 46.2 | 62.9 | 54.4 | 2.09 (0.81 to 3.36) |
| Self-reported health, % | | | | |
| Poor | 6.2 | 5.8 | 6.0 | -0.42 (-0.93 to 0.09) |
| Fair | 14.3 | 15.0 | 15.1 | -0.70 (-1.33 to -0.06) |
| Good or better | 79.5 | 79.2 | 79.1 | 1.02 (0.14 to 1.90) |
| ***Panel B. Heterosexual individuals*** | | | | |
| Insurance coverage, % | 92.8 | 98.8 | 94.2 | 4.25 (4.06 to 4.44) |
| Health care access, % | | | | |
| Have a usual source of care | 88.8 | 94.6 | 92.0 | 1.04 (0.76 to 1.33) |
| Unable to see physician in past year because of cost | 10.3 | 3.7 | 7.5 | -2.13 (-2.78 to -1.48) |
| Received an influenza vaccination in past year | 46.1 | 63.0 | 54.4 | 2.14 (0.82 to 3.46) |
| Self-reported health, % | | | | |
| Poor | 6.1 | 5.7 | 5.9 | -0.35 (-0.86 to 0.17) |
| Fair | 14.1 | 14.8 | 14.9 | -0.63 (-1.28 to 0.01) |
| Good or better | 79.8 | 79.5 | 79.3 | 0.96 (0.07 to 1.85) |
| ***Panel C. Lesbian, Gay, Bisexual, or Another Sexual Minority (LGB+) individuals*** | | | | |
| Insurance coverage, % | 91.9 | 97.4 | 94.0 | 3.59 (2.46 to 4.71) |
| Health care access, % | | | | |
| Have a usual source of care | 88.8 | 93.2 | 91.9 | -0.14 (-1.82 to 1.54) |
| Unable to see physician in past year because of cost | 12.6 | 5.8 | 9.4 | -1.38 (-3.85 to 1.10) |
| Received an influenza vaccination in past year | 51.7 | 62.1 | 58.1 | 1.16 (-1.88 to 4.19) |
| Self-reported health, % | | | | |
| Poor | 7.4 | 7.6 | 7.0 | -0.29 (-2.46 to 1.88) |
| Fair | 17.1 | 17.1 | 18.6 | -2.51 (-4.58 to -0.44) |
| Good or better | 75.5 | 75.3 | 75.2 | 2.44 (-0.87 to 5.75) |

[a] All estimates are unweighted for years 2016-2021.
[b] Column contains the expected mean at age 65 years, which is based on the local linear association between age and the outcome in each row. The expected means contain the counterfactual outcome at age 65 years in the absence of treatment (the expected outcome at 65 years without Medicare).
[c] Adjusted discontinuity estimates are in percentage points.

© 2024 Gavulic KA et al. *JAMA Health Forum.*

**eTable 14. Medicare eligibility age-related discontinuities in coverage, access, and self-reported health by gender identity[a]**

| | Sample Means, % | | Change at age 65 years | |
|---|---|---|---|---|
| | Ages 51-64 | Ages 65-79 | Expected mean, %[b] | Adjusted discontinuity (95% CI)[c] |
| ***Panel A. Full sample*** | | | | |
| Insurance coverage, % | 92.7 | 98.7 | 94.3 | 4.15 (3.96 to 4.35) |
| Health care access, % | | | | |
| Have a usual source of care | 88.8 | 94.5 | 92.0 | 1.10 (0.87 to 1.33) |
| Unable to see physician in past year because of cost | 10.5 | 3.9 | 7.7 | -2.32 (-2.87 to -1.77) |
| Received an influenza vaccination in past year | 46.3 | 62.6 | 54.3 | 2.23 (1.10 to 3.36) |
| Self-reported health, % | | | | |
| Poor | 6.2 | 5.8 | 5.9 | -0.29 (-0.76 to 0.18) |
| Fair | 14.2 | 15.0 | 15.1 | -0.84 (-1.39 to -0.30) |
| Good or better | 79.6 | 79.1 | 79.0 | 1.17 (0.39 to 1.96) |
| ***Panel B. Cisgender Individuals*** | | | | |
| Insurance coverage, % | 92.7 | 98.7 | 94.3 | 4.13 (3.93 to 4.34) |
| Health care access, % | | | | |
| Have a usual source of care | 88.9 | 94.5 | 92.1 | 0.97 (0.72 to 1.21) |
| Unable to see physician in past year because of cost | 10.5 | 3.8 | 7.6 | -2.28 (-2.84 to -1.73) |
| Received an influenza vaccination in past year | 46.4 | 62.6 | 54.3 | 2.24 (1.11 to 3.36) |
| Self-reported health, % | | | | |
| Poor | 6.2 | 5.8 | 5.9 | -0.30 (-0.76 to 0.17) |
| Fair | 14.1 | 15.0 | 15.0 | -0.85 (-1.40 to -0.30) |
| Good or better | 79.7 | 79.2 | 79.1 | 1.19 (0.40 to 1.98) |
| ***Panel C. Transgender and Gender Diverse Individuals*** | | | | |
| Insurance coverage, % | 89.7 | 96.8 | 92.2 | 4.11 (0.82 to 7.39) |
| Health care access, % | | | | |
| Have a usual source of care | 85.7 | 92.3 | 86.8 | 5.16 (-0.87 to 11.18) |
| Unable to see physician in past year because of cost | 14.9 | 8.3 | 12.3 | -2.11 (-7.99 to 3.77) |
| Received an influenza vaccination in past year | 44.1 | 57.0 | 52.8 | -4.44 (-16.14 to 7.26) |
| Self-reported health, % | | | | |
| Poor | 9.7 | 9.4 | 6.9 | 1.40 (-4.25 to 7.06) |
| Fair | 18.3 | 17.5 | 21.8 | -4.01 (-9.14 to 1.11) |
| Good or better | 72.0 | 73.0 | 69.3 | 5.77 (-2.27 to 13.82) |

[a] All estimates are unweighted.
[b] Column contains the expected mean at age 65 years, which is based on the local linear association between age and the outcome in each row. The expected means contain the counterfactual outcome at age 65 years in the absence of treatment (the expected outcome at 65 years without Medicare).
[c] Adjusted discontinuity estimates are in percentage points.

© 2024 Gavulic KA et al. *JAMA Health Forum.*

**eTable 15. Medicare eligibility age-related discontinuities in coverage, access, and self-reported health by sexual orientation, weighted[a]**

| | Sample Means, % | | Change at age 65 years | |
|---|---|---|---|---|
| | Ages 51-64 | Ages 65-79 | Expected mean, %[b] | Adjusted discontinuity (95% CI)[c] |
| ***Panel A. Full sample*** | | | | |
| Insurance coverage, % | 91.0 | 98.2 | 92.9 | 4.95 (4.45 to 5.45) |
| Health care access, % | | | | |
| Have a usual source of care | 87.9 | 94.4 | 91.1 | 1.33 (0.53 to 2.12) |
| Unable to see physician in past year because of cost | 11.8 | 4.5 | 8.3 | -2.08 (-3.29 to -0.86) |
| Received an influenza vaccination in past year | 44.3 | 61.9 | 53.2 | 1.45 (-0.73 to 3.64) |
| Self-reported health, % | | | | |
| Poor | 6.7 | 6.5 | 6.1 | -0.07 (-0.96 to 0.82) |
| Fair | 15.2 | 16.9 | 16.6 | -0.70 (-1.74 to 0.34) |
| Good or better | 78.1 | 76.6 | 76.9 | 1.17 (-0.34 to 2.67) |
| ***Panel B. Heterosexual Individuals*** | | | | |
| Insurance coverage, % | 91.4 | 98.4 | 93.2 | 4.89 (4.33 to 5.46) |
| Health care access, % | | | | |
| Have a usual source of care | 88.1 | 94.7 | 91.3 | 1.49 (0.66 to 2.31) |
| Unable to see physician in past year because of cost | 11.5 | 4.2 | 8.0 | -2.29 (-3.51 to -1.07) |
| Received an influenza vaccination in past year | 44.1 | 62.1 | 53.3 | 1.46 (-0.78 to 3.70) |
| Self-reported health, % | | | | |
| Poor | 6.5 | 6.2 | 5.9 | -0.10 (-0.98 to 0.77) |
| Fair | 14.9 | 16.5 | 16.4 | -0.77 (-1.85 to 0.30) |
| Good or better | 78.6 | 77.3 | 77.6 | 1.11 (-0.41 to 2.62) |
| ***Panel C. Lesbian, Gay, Bisexual, or Another Sexual Minority Identity (LGB+) Individuals*** | | | | |
| Insurance coverage, % | 89.3 | 95.4 | 90.5 | 4.41 (0.32 to 8.49) |
| Health care access, % | | | | |
| Have a usual source of care | 87.6 | 93.1 | 91.3 | -0.33 (-3.37 to 2.72) |
| Unable to see physician in past year because of cost | 13.7 | 7.6 | 12.3 | -3.91 (-7.34 to -0.47) |
| Received an influenza vaccination in past year | 50.7 | 62.2 | 58.6 | 2.36 (-3.55 to 8.28) |
| Self-reported health, % | | | | |
| Poor | 7.8 | 9.0 | 10.6 | -2.65 (-5.96 to 0.65) |
| Fair | 18.3 | 19.5 | 20.9 | -0.97 (-5.56 to 3.63) |
| Good or better | 74.0 | 71.5 | 68.4 | 3.80 (-1.71 to 9.31) |

[a] All estimates are survey weighted.
[b] Column contains the expected mean at age 65 years, which is based on the local linear association between age and the outcome in each row. The expected means contain the counterfactual outcome at age 65 years in the absence of treatment (the expected outcome at 65 years without Medicare).
[c] Adjusted discontinuity estimates are in percentage points and report the RD Honest estimates and bias-adjusted confidence intervals (refer to **Methods** sections for more detail on the statistical model).

© 2024 Gavulic KA et al. *JAMA Health Forum.*

**eTable 16. Medicare eligibility age-related discontinuities in coverage, access, and self-reported health by gender identity, weighted[a]**

| | Sample Means, % | | Change at age 65 years | |
|---|---|---|---|---|
| | Ages 51-64 | Ages 65-79 | Expected mean, %[b] | Adjusted discontinuity (95% CI)[c] |
| ***Panel A. Full sample*** | | | | |
| Insurance coverage, % | 91.0 | 98.2 | 92.9 | 4.95 (4.45 to 5.45) |
| Health care access, % | | | | |
| Have a usual source of care | 87.9 | 94.4 | 91.1 | 1.33 (0.53 to 2.12) |
| Unable to see physician in past year because of cost | 11.8 | 4.5 | 8.3 | -2.08 (-3.29 to -0.86) |
| Received an influenza vaccination in past year | 44.3 | 61.9 | 53.2 | 1.45 (-0.73 to 3.64) |
| Self-reported health, % | | | | |
| Poor | 6.7 | 6.5 | 6.1 | -0.07 (-0.96 to 0.82) |
| Fair | 15.2 | 16.9 | 16.6 | -0.70 (-1.74 to 0.34) |
| Good or better | 78.1 | 76.6 | 76.9 | 1.17 (-0.34 to 2.67) |
| ***Panel B. Cisgender Individuals*** | | | | |
| Insurance coverage, % | 91.0 | 98.2 | 92.9 | 4.95 (4.45 to 5.45) |
| Health care access, % | | | | |
| Have a usual source of care | 87.9 | 94.5 | 91.1 | 1.29 (0.49 to 2.10) |
| Unable to see physician in past year because of cost | 11.8 | 4.4 | 8.3 | -2.05 (-3.27 to -0.83) |
| Received an influenza vaccination in past year | 44.3 | 61.9 | 54.2 | 0.62 (-1.50 to 2.74) |
| Self-reported health, % | | | | |
| Poor | 6.6 | 6.4 | 6.1 | -0.07 (-0.96 to 0.83) |
| Fair | 15.2 | 16.8 | 16.6 | -0.67 (-1.70 to 0.36) |
| Good or better | 78.2 | 76.7 | 77.0 | 1.12 (-0.39 to 2.63) |
| ***Panel C. Transgender and Gender Diverse Individuals*** | | | | |
| Insurance coverage, % | 91.3 | 96.0 | 92.1 | 3.70 (-4.56 to 11.95) |
| Health care access, % | | | | |
| Have a usual source of care | 82.3 | 91.9 | 87.2 | 2.12 (-11.97 to 16.20) |
| Unable to see physician in past year because of cost | 16.1 | 9.4 | 18.0 | -11.05 (-24.64 to 2.54) |
| Received an influenza vaccination in past year | 38.8 | 56.2 | 56.2 | -7.04 (-29.12 to 15.03) |
| Self-reported health, % | | | | |
| Poor | 9.1 | 10.4 | 10.0 | -2.26 (-13.55 to 9.04) |
| Fair | 17.8 | 18.2 | 20.0 | -7.30 (-19.37 to 4.76) |
| Good or better | 73.2 | 71.4 | 70.0 | 9.56 (-5.10 to 24.22) |

[a] All estimates are survey weighted.
[b] Column contains the expected mean at age 65 years, which is based on the local linear association between age and the outcome in each row. The expected means contain the counterfactual outcome at age 65 years in the absence of treatment (the expected outcome at 65 years without Medicare).
[c] Adjusted discontinuity estimates are in percentage points and report the RD Honest estimates and bias-adjusted confidence intervals (refer to **Methods** sections for more detail on the statistical model).

**eTable 17. Robustness of Medicare eligibility age-related discontinuities to alterations in the statistical model[a]**

| | Primary estimate (K=2) | Alternative with K=1 | Alternative with K=4 | Triangular kernel (K=2) |
|---|---|---|---|---|
| ***Panel A. Full sample*** | | | | |
| Insurance Coverage, % | 4.15 (3.96 to 4.35) | 4.23 (4.06 to 4.40) | 4.19 (3.97 to 4.42) | 4.13 (3.94 to 4.32) |
| Health care access, % | | | | |
| Have a usual source of care | 1.10 (0.87 to 1.33) | 1.10 (0.88 to 1.32) | 0.95 (0.69 to 1.20) | 0.79 (0.55 to 1.03) |
| Unable to see physician in past year because of cost | -2.32 (-2.87 to -1.77) | -2.41 (-2.86 to -1.97) | -1.72 (-2.42 to -1.02) | -2.08 (-2.62 to -1.55) |
| Received an influenza vaccination in past year | 2.23 (1.10 to 3.36) | 1.81 (0.90 to 2.71) | 1.45 (0.01 to 2.89) | 1.72 (0.61 to 2.82) |
| Self-reported health, % | | | | |
| Poor | -0.29 (-0.76 to 0.18) | -0.25 (-0.64 to 0.14) | -0.29 (-0.86 to 0.29) | -0.23 (-0.67 to 0.22) |
| Fair | -0.84 (-1.39 to -0.30) | -0.99 (-1.45 to -0.52) | -0.90 (-1.56 to -0.25) | -0.85 (-1.38 to -0.32) |
| Good or better | 1.17 (0.39 to 1.96) | 1.15 (0.50 to 1.81) | 1.17 (0.22 to 2.13) | 1.03 (0.28 to 1.78) |
| ***Panel B. Heterosexual Individuals*** | | | | |
| Insurance Coverage, % | 4.17 (3.98 to 4.36) | 4.22 (4.06 to 4.39) | 4.20 (3.98 to 4.41) | 4.13 (3.94 to 4.32) |
| Health care access, % | | | | |
| Have a usual source of care | 1.14 (0.90 to 1.39) | 1.14 (0.92 to 1.37) | 0.90 (0.61 to 1.18) | 0.84 (0.59 to 1.09) |
| Unable to see physician in past year because of cost | -2.32 (-2.87 to -1.76) | -2.46 (-2.91 to -2.01) | -1.70 (-2.41 to -1.00) | -2.09 (-2.63 to -1.55) |
| Received an influenza vaccination in past year | 2.23 (1.07 to 3.39) | 1.81 (0.88 to 2.74) | 1.61 (0.14 to 3.09) | 1.79 (0.66 to 2.93) |
| Self-reported health, % | | | | |
| Poor | -0.24 (-0.71 to 0.23) | -0.20 (-0.59 to 0.19) | -0.24 (-0.81 to 0.34) | -0.18 (-0.63 to 0.26) |
| Fair | -0.81 (-1.36 to -0.27) | -0.95 (-1.41 to -0.48) | -0.81 (-1.47 to -0.16) | -0.82 (-1.35 to -0.29) |
| Good or better | 1.09 (0.30 to 1.89) | 1.01 (0.35 to 1.68) | 1.09 (0.13 to 2.05) | 0.96 (0.21 to 1.72) |
| ***Panel C. Lesbian, Gay, Bisexual, or Another Sexual Minority Identity (LGB+) Individuals*** | | | | |
| Insurance Coverage, % | 3.55 (2.31 to 4.80) | 3.47 (2.39 to 4.56) | 3.89 (2.47 to 5.31) | 3.38 (2.20 to 4.57) |
| Health care access, % | | | | |
| Have a usual source of care | 0.02 (-1.52 to 1.55) | 0.19 (-1.16 to 1.54) | 0.02 (-1.74 to 1.78) | -0.02 (-1.53 to 1.49) |
| Unable to see physician in past year because of cost | -1.99 (-4.05 to 0.07) | -1.93 (-3.68 to -0.19) | -1.48 (-3.96 to 1.00) | -1.76 (-3.76 to 0.24) |
| Received an influenza vaccination in past year | 1.10 (-1.59 to 3.78) | 1.10 (-1.41 to 3.61) | 0.70 (-2.44 to 3.83) | 1.12 (-1.71 to 3.95) |
| Self-reported health, % | | | | |
| Poor | -0.20 (-2.14 to 1.74) | -0.11 (-1.78 to 1.55) | -0.24 (-2.51 to 2.03) | -0.11 (-1.99 to 1.77) |
| Fair | -2.81 (-4.82 to -0.80) | -2.77 (-4.56 to -0.98) | -2.77 (-5.09 to -0.46) | -2.69 (-4.68 to -0.71) |
| Good or better | 2.60 (-0.40 to 5.60) | 3.35 (0.75 to 5.95) | 1.88 (-1.64 to 5.39) | 2.45 (-0.46 to 5.35) |

[a] All estimates are unweighted. Table assesses the robustness of our regression discontinuity estimates to alterations in the statistical model including relying on alternative bounds on the second derivative of the function that relates each outcome to the running variable and a triangular kernel that places more weight on observations closer to the age-based discontinuity at 65. Please refer to **Methods** sections for more detail on the statistical model.

© 2024 Gavulic KA et al. *JAMA Health Forum.*
