# Supplementary material for: Medicare Eligibility and Changes in Coverage, Access to Care, and Health by Sexual Orientation and Gender Identity
Source: JAMA Health Forum. 2024 Jul 5;5(7):e241756. doi: 10.1001/jamahealthforum.2024.1756 (PMC11227074; doi:10.1001/jamahealthforum.2024.1756)
Supplement: Supplement 2. — Data Sharing Statement [file jamahealthforum-e241756-s002.pdf]

Data Sharing Statement

Gavulic. Medicare Eligibility and Changes in Coverage, Access to Care, and Health by Sexual Orientation and Gender Identity. *JAMA Health Forum*. Published July 05, 2024. doi:10.1001/jamahealthforum.2024.1756

Data
Data available: No

Additional Information
Explanation for why data not available: Data are already publicly accessible. We will provide our analytic code upon request.
